# Machine Learning‐Based Lifetime Prediction of Lithium‐Ion Cells

**DOI:** 10.1002/advs.202200630

**Published:** 2022-08-26

**Authors:** Kai Schofer, Florian Laufer, Jochen Stadler, Severin Hahn, Gerd Gaiselmann, Arnulf Latz, Kai P. Birke

**Affiliations:** ^1^ Research & Development Mercedes‐Benz AG Mercedesstraße 120 70327 Stuttgart Germany; ^2^ Insitute for Photovoltaics ‐ Electrical Energy Storage Systems University of Stuttgart Pfaffenwaldring 47 70569 Stuttgart Germany; ^3^ Institute of Electrochemistry Ulm University Albert‐Einstein‐Allee 47 89081 Ulm Germany; ^4^ Helmholtz Institute for Electrochemical Energy Storage (HIU) Helmholtzstraße 11 89081 Ulm Germany

**Keywords:** aging models, evolutionary algorithms, lifetime prediction, lithium‐ion cells, machine learning

## Abstract

Precise lifetime predictions for lithium‐ion cells are crucial for efficient battery development and thus enable profitable electric vehicles and a sustainable transformation towards zero‐emission mobility. However, limitations remain due to the complex degradation of lithium‐ion cells, strongly influenced by cell design as well as operating and storage conditions. To overcome them, a machine learning framework is developed based on symbolic regression via genetic programming. This evolutionary algorithm is capable of inferring physically interpretable models from cell aging data without requiring domain knowledge. This novel approach is compared against established approaches in case studies, which represent common tasks of lifetime prediction based on cycle and calendar aging data of 104 automotive lithium‐ion pouch‐cells. On average, predictive accuracy for extrapolations over storage time and energy throughput is increased by 38% and 13%, respectively. For predictions over other stress factors, error reductions of up to 77% are achieved. Furthermore, the evolutionary generated aging models meet requirements regarding applicability, generalizability, and interpretability. This highlights the potential of evolutionary algorithms to enhance cell aging predictions as well as insights.

## Graphical abstract

Precise lifetime predictions for lithium‐ion cells are challenging due to their complex aging behavior. Therefore, a machine learning framework based on symbolic regression via genetic programming is introduced here. Its evolutionary generated aging models significantly improve predictive performance compared to established approaches, meet requirements regarding applicability and generalizability, and show the potential of enabling new insights into cell aging.

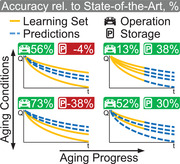

## 1 Introduction

The transformation of the automotive industry towards electric vehicles is inevitable and needs to progress swiftly to reach the goal of zero carbon emissions.^[^ ^1^, ^2^, ^3^ ^]^ Therefore, car manufacturers aim to reduce battery costs, while meeting regulatory requirements and customer needs such as battery lifetimes of about 300 000 km and 15 years under various operating and climatic conditions.^[^ ^4^, ^5^, ^6^, ^7^, ^8^ ^]^ This optimization requires battery lifetime prediction for typical customers based on experimental data. Improvements in its predictive performance considerably contribute to a sustainable mass adoption of electric vehicles as they reduce risks and costs by increasing design competence and development efficiency.^[^ ^8^, ^9^, ^10^, ^11^, ^12^ ^]^ This makes lifetime predictions a crucial part of vehicle development and distinguishes them from remaining useful life predictions, which focus on operational safety by regular on‐board estimations.^[^ ^13^ ^]^

Such a lifetime‐oriented design of batteries and their operating strategies requires precise aging models. However, developing these models is extremely challenging since lithium‐ion cell degradation is very complex. In addition, the high testing effort only allows for relatively small datasets.^[^ ^9^, ^14^, ^15^ ^]^ Cell capacity fade characterized by the state of health (*SoH*_C_) reduces the range of electric vehicles. It results from calendar aging occurring at all times and cycle aging due to cell operation.^[^ ^16^ ^]^ Therefore, lifetime prediction needs to extrapolate over time (*t*) and energy throughput (*ETP*), respectively. Furthermore, extra‐ and interpolation over stress factors of both aging types are required in order to consider differences in user behavior. In summary, cell aging models need to describe the possibly interdependent influence of stress factors on capacity fade based on relatively small datasets and predict long‐term aging behavior even for untested storage and operating conditions.

The challenge for these regression tasks is generalization—the ability to predict the response to previously unseen inputs. This robustness requires models to fit the trend in the data available for learning without describing the noise. While increasing model complexity makes precise fits on learning data more likely, it also heightens the risk of low generalizability by not describing the underlying trends. Consequently, lifetime prediction is a multi‐objective optimization task, which has to consider model accuracy and complexity.^[^ ^17^, ^18^ ^]^

Modeling approaches for lifetime prediction range from physicochemical to empirical.^[^ ^12^ ^]^ Physicochemical models usually simulate single aging mechanisms.^[^ ^10^ ^]^ Despite expanding the knowledge about cell aging, their long‐term predictions are adversely affected by an insufficient consideration of the interdependency of aging mechanisms and an effortful parameterization and computation.^[^ ^9^, ^19^ ^]^ In contrast, empirical models are data‐driven and do not require knowledge about aging mechanisms. Common issues are low interpretability and generalizability.^[^ ^14^, ^19^ ^]^ While recently proposed machine learning approaches such as by Severson et al.^[^ ^14^ ^]^ reduce these disadvantages, the black box models resulting from common machine learning algorithms cannot substantially enhance the understanding of cell aging.^[^ ^20^ ^]^ Therefore, semi‐empirical models, a compromise between physicochemical and empirical approaches, are still considered state‐of‐the‐art for predicting the lifetime of lithium‐ion cells. These models usually consist of a single function that is derived from the main degradation mechanisms and fitted to aging data.^[^ ^8^, ^9^ ^]^ However, these pre‐defined functions impair model generalizability due to limited knowledge about degradation mechanisms.^[^ ^21^, ^22^ ^]^

To overcome the limitations resulting from the current understanding of cell aging, we introduce evolutionary algorithms into lifetime prediction for lithium‐ion cells. The use of symbolic regression via genetic programming as core of a machine learning framework enables a new method of model development. It combines the advantages of machine learning methods, by inferring model structure and parameters from aging data without requiring domain knowledge, and semi‐empirical approaches, by providing potentially interpretable mathematical functions, comparable to state‐of‐the‐art cell aging models. This novel approach reliably develops aging models with high predictive accuracy and low complexity from randomly generated initial models by performing multiple evolutionary processes.

We evaluate this method with case studies, which represent common tasks of lifetime prediction. In Section 2.1, we present the data used for this investigation. It comprises two typical aging experiments with automotive lithium‐ion pouch‐cells (graphite/NMC): While calendar aging was examined with 54 high‐energy cells, cycle aging was performed with 62 high‐power cells. This is followed by an introduction of established modeling concepts in Section 2.2 and an overview of the principles behind our method in Section 2.3. The subsequent comparison with state‐of‐the‐art lifetime prediction models and machine learning approaches in Section 3 reveals significant improvements of predictive accuracy by our algorithm for both examined cell and aging types. Achieving competitive results in each category with a unified modeling approach underlines the versatility and robustness of our method.

Further evaluation of the evolutionary generated aging models focuses on applicability, generalizability, and interpretability. Section 4.1 proves their applicability, which enables a direct replacement of currently used lifetime prediction models while providing the same functionality. The in‐depth analysis of model robustness in Section 4.2 reveals limits of their generalizability for datasets not designed for machine learning. However, these are overcome by a hybrid modeling approach. Finally, Section 4.3 shows their potential regarding interpretability by comparing the structure of the evolutionary generated models with established theories and by using our algorithm to improve already existing models. This highlights the capability of evolutionary algorithms to enhance not only predictions but also insights into complex problems such as cell aging.

For the interested reader, Methods and the Supporting Information contain more details regarding aging data, machine learning framework, and each investigation.

## 2 Data and Modeling Approaches for Lifetime Prediction

### 2.1 Cell Aging Data

Calendar and cycle aging are usually investigated and modeled separately and subsequently combined via superposition.^[^ ^23^ ^]^ To allow for an exhaustive comparison of modeling approaches, data is used from experiments with quality and scope representative for vehicle development.

Calendar aging is influenced by the stress factors state of charge (*SoC*) and temperature (*T*) which are generally agreed not to be interdependent.^[^ ^23^ ^]^ Thus, calendar aging experiments usually vary each factor with the other held constant to enable individual fits of each stress factor's complex dependencies by semi‐empirical models.^[^ ^23^, ^24^ ^]^ As described in Methods, Hahn et al.^[^ ^8^ ^]^ applied this approach in their extensive investigation of 54 high‐energy automotive lithium‐ion pouch‐cells (Figure S1, Supporting Information). In 280 days of effective aging, they obtained well‐resolved *C*/10 (cf. [25]) capacity data. The different storage conditions resulted in a wide variety of *SoH*_C_ ranging from 96% to 78% at the end of experiment (Figure S2, Supporting Information). However, since the experiment was designed for semi‐empirical models, applying machine learning methods is expected to be challenging.

In contrast, cycle aging is a multivariate problem for which the interdependency of several stress factors is not yet fully understood.^[^ ^26^ ^]^ Temperature (*T*), minimum and maximum state of charge (*SoC*_min/max_), charging power (*P*_ch_) and the ratio between charge depleting and sustaining driving mode (*EV*_ratio_) are considered especially relevant.^[^ ^15^, ^19^, ^22^, ^27^ ^]^ Therefore, this work utilizes a statistically designed experiment previously published by Stadler et al.^[^ ^28^ ^]^: The stress factors were varied systematically from a common center point (Figure S3, Supporting Information). This design is particularly well suited for data‐driven modeling approaches. In total, 62 high‐power automotive lithium‐ion pouch‐cells were cycled for roughly 2 years with regular capacity check‐ups. About 1500 *C*/10 capacity values are available, characterizing a broad range of aging conditions, which resulted in *SoH*_C_ between 97% and 90% at the end of experiment (Figure S4, Supporting Information). For more details, see Methods.

### 2.2 Established Modeling Concepts for Lifetime Prediction

Our lifetime prediction framework aims to combine the advantages of machine learning and semi‐empirical models. A thorough analysis of its predictive performance requires a comparison against the best, established approaches for lithium‐ion cell lifetime prediction applicable to the available aging data. For this, the main stress factors of calendar and cycle aging as well as corresponding state‐of‐the‐art modeling concepts are discussed. In Section 3, we benchmark our method against the best‐in‐class models of this wide array of highly competitive approaches.

For calendar aging, degradation is mainly attributed to a growing solid electrolyte interface (SEI).^[^ ^29^, ^30^ ^]^ The theories by Broussely et al.^[^ ^31^ ^]^ and Ploehn et al.^[^ ^32^ ^]^ predict the well‐known $\sqrt{t}$ relationship for passivating SEI growth and thus capacity loss over time. The addition of an Arrhenius relationship for temperature dependency and a similar exponential dependency on *SoC* inspired by the Tafel‐equation prevails in the literature.^[^ ^8^, ^11^ ^]^ The resulting semi‐empirical models describe aging as a function of time as well as distributions of temperatures and storage *SoC*s.^[^ ^8^ ^]^ A wealth of research explores different time^[^ ^10^, ^11^, ^12^, ^33^, ^34^ ^]^ and *SoC* ^[^ ^19^, ^35^ ^]^ dependencies. Recent publications^[^ ^24^, ^36^ ^]^ suggested an anode potential dependency as implemented by Schimpe et al.^[^ ^23^ ^]^ The most competitive modeling approaches are summarized in Table S1, Supporting Information, and considered for a benchmark analysis.

In comparison, cycle aging models are usually more complex as more factors are relevant. Aging is often cumulated over *ETP* or cycle number.^[^ ^15^ ^]^ Temperature and depth of discharge (*DoD*) are widely considered as main stress factors.^[^ ^15^, ^19^, ^22^, ^37^, ^38^ ^]^ The effect of current levels and profiles is discussed more controversially and seems to be cell dependent.^[^ ^19^, ^37^, ^39^ ^]^ Many try to apply empirically determined relationships such as the Arrhenius temperature dependency, leading to a large diversity in investigated stress factors and resulting cycle aging models.^[^ ^10^, ^19^, ^27^, ^39^ ^]^ Since most of these models are designed for a specific dataset and cannot be applied to other datasets, a simplistic power law $SoH_{C}=p_{1}\cdot ETP^{p_{2}}$ with the fitting parameters *p*_i_ is used as strong benchmark for extrapolations over *ETP*. Another empirical approach, which in a similar form was already shown to work adequately on the available data,^[^ ^28^ ^]^ additionally enables predictions over operating conditions: Each cell's aging trend is fitted with $SoH_{C}=p_{1}\cdot ETP^{0.7}$ in combination with multivariate regression to parameterize an operating condition dependent model of *p*_1_ (*T*, *SoC*_min_, *SoC*_max_, *P*_ch_, *EV*_ratio_). Both approaches are chosen for further evaluation (Table S2, Supporting Information).

Furthermore, a comprehensive selection of established machine learning approaches is investigated for comparison. Two forms of linear regression (lasso and ridge) are considered, representing the group of white box models, cf. refs. [40, 41, 42]. Additionally, the ensemble learning techniques extreme gradient boosting and random forest regression are inspected due to their state‐of‐the‐art results on structured data.^[^ ^43^, ^44^ ^]^ Gaussian process regression is chosen since its known ability to generalize on small datasets.^[^ ^45^ ^]^ For completion, a multi‐layer perceptron neural network and a simple and robust alternative approach for symbolic regression, fast function extraction, are included.^[^ ^46^, ^47^ ^]^ Details about applied software and hyperparameter optimization are available in Table S3, Supporting Information.

### 2.3 Genetic Programming‐Based Machine Learning Framework

In order to infer model structure and parameters without requiring prior domain knowledge, this work implements multi‐gene symbolic regression via genetic programming as core of a machine learning framework for lifetime prediction. In this section, we aim to provide a high‐level overview of the principles of our framework and refer to Methods and the Supporting Information for more detailed information.

**Figure 1:**
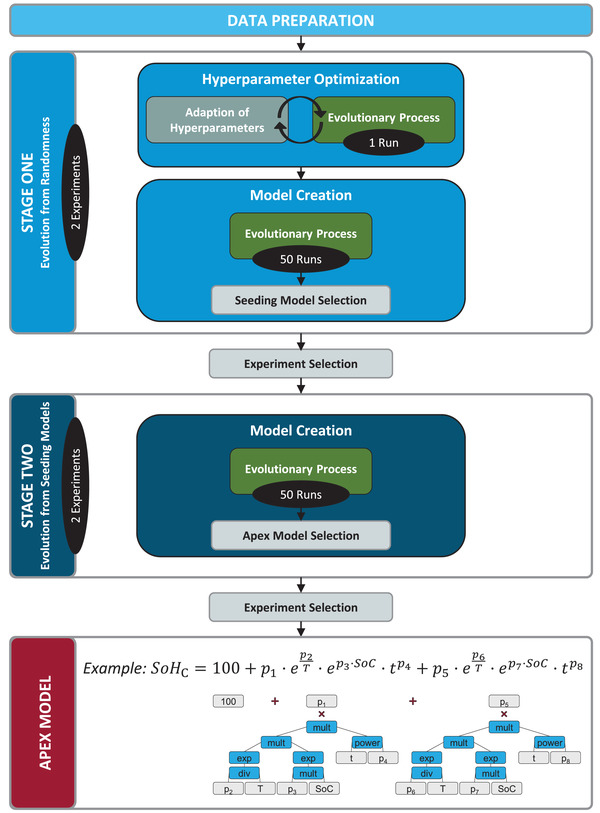
Framework structure for the evolutionary generation of lifetime prediction models: After Data Preparation as interface to cell aging data bases, aging model structures and parameters are inferred without requiring prior domain knowledge by multi‐gene symbolic regression via genetic programming as evolutionary process. This core is embedded into a multi‐stage structure ensuring predictive accuracy and reliability of the framework's output—the apex model. As exemplified by a typical semi‐empirical aging model, these models are represented as trees.

Genetic programming is a subclass of evolutionary algorithms, which are stochastic optimization algorithms inspired by Darwin's theory of biological evolution.^[^ ^21^, ^48^ ^]^ Their common concept is the generation‐wise evolution of a population consisting of possible solutions to the optimization problem—the individuals.^[^ ^48^ ^]^ In multi‐gene symbolic regression, these individuals are mathematical equations typically represented as one or more scaled trees and a bias term.^[^ ^20^ ^]^ Each tree consists of numerical constants and variables as external nodes and mathematical operations as internal nodes.^[^ ^48^ ^]^ This concept is exemplarily shown for a typical semi‐empirical calendar aging model in **Figure** **1**.

For its symbolic regression core, this work uses a modified version of the GPTIPS 2 framework by Searson et al.^[^ ^20^ ^]^ It starts with the random creation of an initial population and the subsequent evaluation of each individual's fitness.^[^ ^21^, ^48^, ^49^, ^50^ ^]^ In our application, a fitness function assigns each of the various initial aging models a numerical value representing their generalizability, that is, a combination of model accuracy and complexity. This is followed by a generational loop of evolution, which consists of parent selection, reproduction, fitness evaluation, and survivor selection.

Each iteration of this loop starts with the fitness‐based selection of individuals for reproduction. Stochastic choice determines if recombination, manipulation, or cloning is applied to these parents in order to produce offspring. After evaluating the offspring's fitness, each iteration is concluded by selecting surviving individuals based on their age and fitness to form the successive generation. The variation by reproduction combined with fitness‐based selection drives this optimization process.^[^ ^21^, ^48^, ^49^, ^50^ ^]^ In the case of lifetime prediction models, this enables an evolution from random combinations of constants, variables, and mathematical operations towards a pool of generalizable aging models. A model is considered generalizable if it is capable to correctly predict the aging behavior of the investigated cell and aging type for conditions not utilized in the learning process. The generational loop is iterated until a termination criterion is satisfied and the final generation's best individuals are returned.^[^ ^21^, ^48^, ^49^, ^50^ ^]^ In our version, only reaching a specific number of generations terminates the evolutionary process. This is one of many hyperparameters, which define the evolutionary process and require optimization by an additional algorithm to ensure an efficient and reliable development of generalizable models. The evolutionary process is summarized in Figure S5, Supporting Information. For a detailed description of all its parts and the adjustments implemented in the modified GPTIPS 2 framework, we refer to Note S1.1, Supporting Information.

We embedded this symbolic regression core into a machine learning framework as shown in Figure 1 and further explained in Methods. This framework enables the automated inference of reliable lifetime prediction models from typical aging data. Its structure mitigates the risk of non‐deterministic evolutionary processes and thereby provides a robust and efficient method for an unbiased development of aging models. The framework consists of "Data Preparation", "Stage One", and "Stage Two". Data Preparation serves as interface to cell aging databases by transforming raw aging data into a training and validation set used for the learning process as well as a test set reserved for a‐posteriori performance evaluations. Stage One performs multiple separate evolutionary runs starting from randomly generated initial populations. After optimizing hyperparameters, which define the evolutionary algorithm's behavior, Stage One creates a pool of promising seeding models. Stage Two uses these models as starting point of its evolutionary processes to improve predictive accuracy and reliability. Stage Two concludes by selecting one well generalizable model as final output, the apex model. To further attenuate the risk resulting from non‐deterministic algorithms, each stage is evaluated in two independent experiments. After each stage, a selection mechanism aims at choosing the more suitable experiment.

## 3 Performance Comparison of Modeling Approaches

The modeling approaches are evaluated in case studies to allow for an exhaustive and unbiased examination of predictive performance. Each case study consists of various test cases, which represent the main tasks of lifetime prediction for calendar and cycle aging. For this, the aging data is prepared as explained in Methods and split into different training, validation, and test sets.

**Figure 2:**
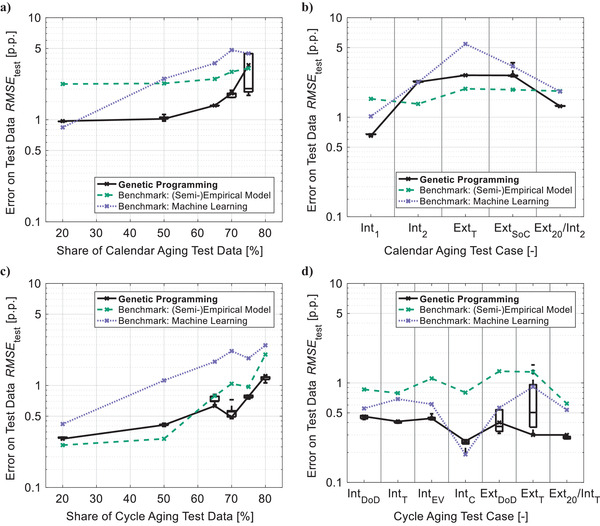
Results of the case studies: Typical tasks of lifetime prediction are investigated with a) extrapolation of calendar aging over storage time, b) prediction of calendar aging over storage conditions, c) extrapolation of cycle aging over energy throughput, and d) prediction of cycle aging over operating conditions. A detailed description of all test cases is available in Figures S6–S12 and Tables S4 and S5, Supporting Information. The genetic programming framework's predictive error regarding *SoH*_C_ in percentage points (p.p.) is compared against state‐of‐the‐art lifetime prediction and machine learning approaches. For genetic programming, boxes represent the selection risk via each test case's ten highest ranked models—with a central mark for median, bottom/top edges for 25^th^/75^th^ percentiles, and whiskers for best/worst model.

The calendar aging case study examines extrapolation over storage time (**Figure** **2**a) and predictions over storage conditions (Figure 2b): While test cases Cal Int_1_ and Cal Int_2_ investigate interpolation between storage conditions, Cal Ext_T_ and Cal Ext_SoC_ look into extrapolation over *T* and *SoC*, respectively. For extrapolation over time, the test set's share—and therefore the predictive challenge—is increased stepwise from Cal Ext_20_ with 20% to Cal Ext_75_ with 75%. Extrapolation over time and interpolation between storage conditions are combined in Cal Ext_20_/Int_2_. Similarly, the cycle aging case study examines extrapolation over *ETP* (Figure 2c) and predictions over operating conditions (Figure 2d): Interpolation between operating conditions is investigated by Cyc Int_DoD_, Cyc Int_T_, and Cyc Int_EV_ with focus on the influence of *DoD*, *T*, and *EV*_ratio_, respectively. Additionally, Cyc Int_C_ interpolates the experiment's center point. Extrapolations over *DoD* and *T* are investigated by Cyc Ext_DoD_ and Cyc Ext_T_, respectively. Furthermore, Cyc Ext_20_‐Ext_80_ analyze extrapolation over *ETP* with increasing predictive challenge. The test case Cyc Ext_20_/Int_T_ combines Cyc Ext_20_ and Cyc Int_T_. A detailed display of all test cases’ data partitions is available in Figures S6–S12, Supporting Information. The resulting predictive tasks and shares of datasets are summarized in Tables S4 and S5, Supporting Information.

For performance analysis, all modeling approaches are evaluated by their root mean square error *RMSE*_test_ on the test data of each test case. Since test data is not used for learning and thus a previously unseen input, the *RMSE*_test_ represents a model's ability to predict aging for a specific interpolation or extrapolation task. A definition of this metric is available in Methods. In Figure 2, the predictive errors of our framework are visualized by crosses for apex models and boxes representing the selection risk. Note the logarithmic scale, which allows for a detailed evaluation of predictions with high accuracy while also including results of far less precise models. For most test cases, the combination of multi‐stage algorithm structure and elaborate selection mechanisms ensures an almost negligible selection risk and thus high reliability. Exceptions are extreme extrapolations over storage time (Cal Ext_75_ with a test data share of 75%, Figure 2a) and cycle aging stress factors (Cyc Ext_DoD_/Cyc Ext_T_, Figure 2d) due to a lack of information in the learning data. The results of the framework are compared with the state‐of‐the‐art, which is determined by a benchmark analysis based on the findings of Section 2.2 and described in Note S2, Supporting Information. According to this analysis, the calendar aging model by Belt et al.^[^ ^33^ ^]^ and the cycle aging model inspired by Stadler et al.^[^ ^28^ ^]^ are selected as best applicable (semi‐)empirical models. Extreme gradient boosting (see ref. [44]) represents established machine learning approaches. Detailed results are available in Figures S13–S15 and Tables S1–S3, Supporting Information.

For a compact quantification of predictive performance, each test case is evaluated by comparing the predictive errors *RMSE*_test_ of apex model and benchmark approaches (see Figure 2) resulting in a relative error. For similar test cases, an average of these relative errors is calculated as summarized in Tables S6 and S7, Supporting Information.

**Figure 3:**
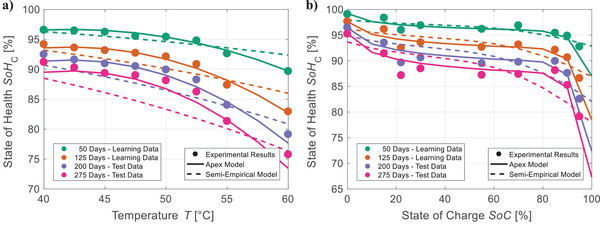
Representation of stress factors: For test case Cal Ext_50_, the experimental results are compared with the predictions of the evolutionary generated apex model and the benchmark semi‐empirical model for the available learning data (0–140 days of storage) and test data (141–180 days of storage). The influence of a) temperature and b) *SoC* is investigated for *SoC* = 85% and *T* = 50 °C, respectively. The evolutionary generated model achieves a significantly more accurate description of the physical correlations than the state‐of‐the‐art.

The comparison against (semi‐)empirical benchmark models reveals average improvements of 38% and 13% for extrapolation over storage time (Figure 2a) and *ETP* (Figure 2c), respectively. In addition, moderate interpolation (Cal Int_1_) and combined prediction (Cal Ext_20_/Int_2_) of calendar aging (Figure 2b) are improved by 58% and 30%, respectively. These advancements result from a significantly better consideration of stress factors as exemplarily shown for Cal Ext_50_ in **Figure** **3**. The apex model of this test case describes the dependence of *SoH*_C_ on temperature and *SoC*—the main stress factors of calendar aging—drastically more accurate on learning as well as test data than the semi‐empirical benchmark model. This improved representation of stress factors is further highlighted by the test cases investigating the complex prediction over cycle aging conditions (Figure 2d): Our algorithm achieves $\bar{RMSE}$ reductions of 56% for interpolation (Cyc Int_DoD_/Cyc Int_T_/Cyc Int_EV_/Cyc Int_C_), of 73% for extrapolation (Cyc Ext_DoD_/Cyc Ext_T_), and of 52% for combined aging prediction (Cyc Ext_20_/Int_T_). However, for extrapolation over storage conditions (Cal Ext_T_/Cal Ext_SoC_) and extreme interpolation (Cal Int_2_) (Figure 2b), the $\bar{RMSE}$ is increased by 38% and 66%, respectively. Most likely, this is caused by insufficient information in the learning data and can potentially be avoided without affecting testing effort by a design of experiment optimized for machine learning.

The improvements by our algorithm are even more consistent in comparison with the benchmark machine learning approach. Our framework reduces the $\bar{RMSE}$ for extrapolations over storage time (Figure 2a) and *ETP* (Figure 2c) by 38% and 57%, respectively. In addition, it achieves much higher reliability for predictions over stress factors: The average predictive accuracy for interpolation and extrapolation over aging conditions as well as combined prediction is improved by 18%, 35%, and 29% for calendar aging (Figure 2b) and by 12%, 48%, and 44% for cycle aging (Figure 2d).

The performance improvements by our approach enable not only predictions that are more accurate but also a drastically reduced testing effort. For example, the genetic programming framework still achieves 19% more precise extrapolations over storage time than the state‐of‐the‐art while using 140 days instead of 280 days for learning. Additionally, more sophisticated cell operating strategies become possible by an improved prediction of the influence of different operating and storage conditions on cell aging.

## 4 Evaluation of Evolutionary Generated Aging Models

To realize these advantages in vehicle development, the apex models need to meet requirements regarding applicability and generalizability. Furthermore, they have to show the potential of interpretability in order to enable new insights into cell aging. Therefore, the following evaluation of evolutionary generated aging models focuses on these three aspects.

### 4.1 Model Applicability

In vehicle development, load collectives of operational profiles are frequently used for lifetime prediction.^[^ ^51^ ^]^ Since this removes the chronology from aging data, path independency is usually required for lifetime prediction models to be applicable. While path independency for calendar aging was proven experimentally regarding *T* and *SoC*,^[^ ^52^ ^]^ the literature indicates current related path dependencies for cycle aging.^[^ ^53^ ^]^

Inspired by Su et al.^[^ ^52^ ^]^, we examine path independency by generating five random chronological variations of a hypothetical calendar aging load collective for each influence factor. While varying one factor, the others remain constant. A repetition of these load profiles ensures equal collective loads at 0%, 50%, and 100% testing progress. Degradation is computed by switching between aging curves for constant conditions (Figure S16, Supporting Information).

**Figure 4:**
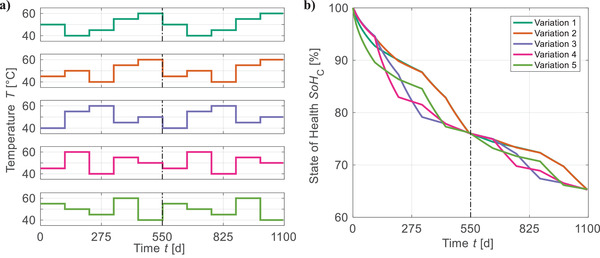
Verification of path independency for evolutionary generated lifetime prediction models: As exemplarily shown for the temperature dependency of the apex model of Cal Ext_20_, a) five random chronological variations of a hypothetical load collective are generated for each stress factor and b) the aging trends converge to the same *SoH*_C_ at points of equal load ($\hat{=}$0/550/1100 days) for all investigated test cases.

As exemplarily shown for the temperature dependency of the apex model of Cal Ext_20_ in **Figure** **4**, all aging trends converge to the same *SoH*_C_ at points of equal load. This commutative behavior emerges for all stress factors and investigated test cases. Thus, the models generated by our framework can be considered path independent and utilized for analyzing load collectives.

### 4.2 Model Generalizability

**Figure 5:**
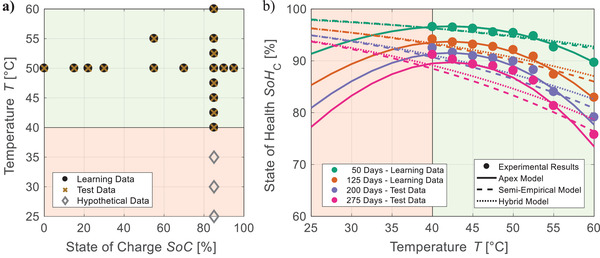
Generalizability of calendar aging models: a) Degradation is simulated for hypothetical data at lower temperatures than the available learning data (0–140 days of storage) and test data (141–180 days of storage). b) For test case Cal Ext_50_, the experimental results are compared with the predictions of the evolutionary generated apex model, the benchmark semi‐empirical model, and a hybrid of both.

The results on the available data indicate that our algorithm can flexibly develop well‐generalizable models for various use cases: Each test case's apex model represents general aging trends without describing noise (Figures S17 and S18, Supporting Information). For a more detailed analysis of generalizability, degradation is simulated for hypothetical data beyond the available data (**Figure** **5**a) and compared with commonly accepted physicochemical relationships, such as the Arrhenius temperature dependency.^[^ ^8^ ^]^ As shown in Figure 5b, the apex model for Cal Ext_50_ accurately fits the available learning and test data. However, the opposing trend of the Arrhenius dependency‐based semi‐empirical model for temperatures below 40 °C (hypothetical data) proves that extrapolation over aging conditions far beyond learned correlations can be challenging.

To ensure meaningful predictions for all aging conditions, we propose a hybridization of evolutionary generated and semi‐empirical models by utilizing our models to expand the learning data for parameterizing semi‐empirical models: The apex model of the investigated test case predicts aging for multiple artificial grids. The resulting data is used to train the semi‐empirical benchmark model (Figure S19, Supporting Information). This enables reasonable predictions for all conditions (Figure 5b) and thus ensures a generalizable output by our framework even in cases of insufficient information for the learning process.

**Figure 6:**
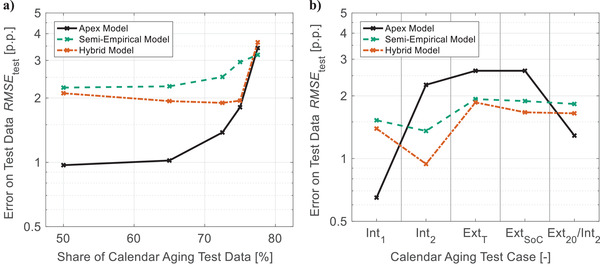
Predictive performance of hybridization: The predictive errors regarding *SoH*_C_ in percentage points (p.p.) of evolutionary generated models, the semi‐empirical benchmark model, and a hybrid modeling approach are compared for typical tasks of lifetime prediction—a) extrapolation of calendar aging over storage time and b) prediction of calendar aging over storage conditions. A detailed description of all test cases is available in Figures S6–S8 and Table S4, Supporting Information.

The results for the test cases Cal Ext_T_, Cal Ext_SoC_, and Cal Int_2_ in **Figure** **6**b support this observation. While the original apex models provide a lower predictive accuracy than the semi‐empirical benchmark model for these test cases (see also Figure 2b), the hybrid models improve upon both approaches. For the remaining test cases in Figure 6a,b, the hybrid models are not as precise as the original apex models but still significantly better than the state‐of‐the‐art. In summary, the hybrid approach does not fully exploit our algorithms' potential but drastically increases its robustness.

The presented hybridization approach successfully compensates a lack of information in the learning data by considering domain knowledge after finishing the evolutionary model generation. Integrating domain knowledge directly into the evolutionary process could achieve the same effect while compromising less on maximum predictive performance. Furthermore, for cycle aging, our apex models extrapolate significantly better over aging conditions than for calendar aging (see Figure 2b,d). Most likely, this results from a more suitable design of experiment. Therefore, further enhancements in generalizability are expected from experiments optimized for machine learning.

### 4.3 Model Interpretability

Our algorithm shows enormous potential regarding physical interpretation: The calendar aging apex models prevalently consider $\sqrt{t}$ dependencies as shown in Note S3.1, Supporting Information. This conforms with the theories of Broussely et al.^[^ ^31^ ^]^ and Ploehn et al.^[^ ^32^ ^]^ despite derivation solely from data. These apex models result from independent evolutionary processes, which start with randomly generated initial models; nonetheless they all include the same commonly accepted theoretical dependency. This highlights the potential of our method to improve the understanding of cell aging by uncovering currently unknown relations. However, it has to be noted that so far most evolutionary generated models are rather complex and partially feature uninterpretable correlations. This can likely be overcome by considering domain knowledge in the evolutionary process. Promising approaches are constraints, seeding of semi‐empirical models, or the definition of physically motivated building blocks for internal nodes.

First investigations with seeding semi‐empirical models in initial populations of Stage One with proportions ranging from 20% to 100% for calendar aging test cases are explained in Note S3.2, Supporting Information. They revealed a similar predictive accuracy compared to random initialization but also an increased selection risk (Figure S20, Supporting Information). This can result from confining the search space too rapidly to local optima due to seeding. However, this investigation's apex models resemble the seeded models and thus highlight the opportunity of enhancing existent theories with our algorithm. Consequently, future work towards a better integration of domain knowledge could facilitate new insights into the influence of various aging conditions on cell aging.

## 5 Conclusion

With our framework, we introduce symbolic regression via genetic programming as a promising approach for lifetime prediction of lithium‐ion cells. Our framework applies evolutionary concepts to generate aging models from randomness. For this, it uses conventional calendar and cycle aging data of automotive lithium‐ion pouch‐cells (graphite/NMC) for conditions representing typical customer behavior. Case studies, which investigate relevant tasks of lifetime prediction, reveal significantly enhanced predictive accuracy compared to state‐of‐the‐art semi‐empirical models and common machine learning approaches. While the investigated datasets are large for cell aging, they are unfavorable for machine learning. Still, compared to the best applicable semi‐empirical models predictive accuracy is increased by 38% and 13% on average for extrapolations over storage time and *ETP*, respectively. For predictions over other stress factors, improvements of up to 77% are achieved. This is possible due to a better representation of the interdependent influence of various stress factors. As our data‐driven modeling concept also meets requirements regarding applicability and generalizability, it enables more efficient battery development and more sophisticated operating strategies. Furthermore, the interpretability of the evolutionary generated models allows our novel approach to enhance the understanding of cell aging. In general, this work highlights the potential of data‐driven modeling not only for aging of lithium‐ion cells but also for other complex problems currently limited by insufficient domain knowledge.

## 6 Methods

### 6.1 Cell Aging Experiments

The aging data used in this work was published in detail by Hahn et al.^[^ ^8^ ^]^ for calendar aging and Stadler et al.^[^ ^28^ ^]^ for cycle aging. They investigated the automotive lithium‐ion pouch‐cells described in **Table** **1**. Both experiments were performed in climate chambers monitored to be within ±1 K of the target temperatures.

**Table 1:** Properties of the investigated automotive lithium‐ion pouch‐cells

Properties	Calendar aging experiment	Cycle aging experiment
Amount tested	54 cells	60 cells
Type	High energy	High power
Negative electrode	Graphite	Graphite
Positive electrode	Li(Ni_1/3_Mn_1/3_Co_1/3_)O_2_	Blend of Li(Ni_0.__6_Mn_0.__2_Co_0.__2_)O_2_ and Li(Ni_1/3_Mn_1__/3_Co_1__/3_)O_2_
Nominal capacity *C*_N_ [Ah]	50.8	43
Minimum cell voltage *V*_cell,min_ [V]	3.0	2.5
Maximum cell voltage *V*_cell,max_ [V]	4.2	4.2

During both experiments, periodic checkups were performed at 25 °C beginning with three cycles of constant‐current constant‐voltage (CC–CV) charge to *V*_cell,max_ (1*C* constant current, *C*/20 cut‐off current) and CC discharge to *V*_cell,min_ (1*C* constant current) with the goal of equilibrating the cells. Following another CC–CV charge to *V*_cell,max_ and a pause of 1 h, the cells were discharged with *C*/10 to *V*_cell,min_ to determine the *C*/10 capacity degradation used in this work. This measure was shown to be largely unaffected by overhang effects,^[^ ^54^ ^]^ which often plague aging experiments.^[^ ^22^ ^]^

As displayed in Figure S1, Supporting Information, the accelerated calendar aging experiment was designed to examine the influence of storage temperature and *SoC* within 280 days of effective aging: The temperature was varied between 40 and 60 °C at *SoC* = 85% with four cells per condition. Furthermore, two cells each were tested for *SoC*s ranging from 0% to 100% at 50 °C. Two additional cells were aged at *SoC* = 55% and 55 °C.

The cells were mounted into cell holders at their pouch seams allowing for unconstrained cell expansion. While the cells were aged in open circuit condition, they were recharged daily by CC–CV charging to their storage voltage with 0.06*C* constant current and 0.004*C* cut‐off current. The storage voltage was always set before exposing the cells to the storage temperature.

The cycle aging experiment was planned according to the statistical concept of central composite design using the software Minitab. Five especially relevant stress factors were selected based on literature review and expert knowledge. Starting from a common center point, these factors were varied between five evenly distributed levels (**Table** **2** and Figure S3, Supporting Information). While the center point was investigated with eight cells, the remaining 26 variations of operating conditions were examined with two cells each.

**Table 2:** Design of experiment matrix for cycle cell aging: The stress factors temperature (*T*), minimum and maximum state of charge (*SoC*_min/max_), charging power (*P*_ch_), and ratio between charge depleting and sustaining driving mode (*EV*_ratio_) were statistically varied between five evenly distributed levels. Each operating condition was investigated with multiple cells

Factor	Minimum	Low	Center	High	Maximum
*T* [°C]	11	21	28	41	50
*P*_ch_ [W]	8	72	136	200	264
*SoC*_min_ [%]	21	25	29	33	37
*SoC*_max_ [%]	81	85	90	95	100
*EV*_ratio_ [%]	20	40	60	80	100

All cells were tested in a setup that maintains a constant force of 1.1 kN. Between check‐ups, the cells were continuously cycled for 14 days under constant chamber temperature with a charge depleting and a charge sustaining profile to mimic real life usage of plug‐in hybrid electric vehicles. Every cycle consisted of charging with a constant‐power constant‐voltage (CP–CV) procedure with *P*_ch_ to *SoC*_max_ and discharging with the charge depleting profile to *SoC*_min_. Afterwards, the charge sustaining profile was repeated until the specified *EV*_ratio_ (ratio of *ETP* in charge depleting mode vs entire *ETP* of this cycle) was met.

### 6.2 Machine Learning Framework

The machine learning framework consists of the blocks Data Preparation, Stage One, and Stage Two. Furthermore, selection mechanisms are required to identify the best models out of a variety of potential solutions. This structure is summarized in Figure 1 and subsequently explained in detail.

#### 6.2.1 Data Preparation

Data Preparation serves as interface to cell aging databases. It transforms raw aging data into the specified format before partitioning it into training, validation, and test set. For this work's data, prioritization of aging conditions investigated with different amounts of cells was avoided by averaging information of selected cells as shown in Figures S2 and S4, Supporting Information. Furthermore, each aging condition's information was linearly interpolated to ensure equal representation independent of overall storage time or *ETP*. The selected sample rate for cycle aging (50 data points equally distributed over each test's progress) was significantly lower than for calendar aging (250 data points) since fewer measurements per test series were available and excessive interpolation was expected to be ineffective. The data was separated into predictor and response variables. Both investigated case studies utilize the *SoH*_C_ as response variable. As shown by Lewerenz et al.^[^ ^54^ ^]^, anode overhang can effect an initial rise in capacity depending on the storage *SoC* prior and during calendar cell aging tests. This effect is reversible and thus detrimental to modeling cell aging.^[^ ^8^ ^]^ To decrease its influence, *SoH*_C_ is defined as ratio of measured capacity *C*_M_ to maximum measured capacity *C*_M,max_:

(1) $$SoH_{C}=\frac{C_{M}}{C_{M,\max}}$$

For the calendar aging case study, storage time, *T*, and *SoC* represented as cell voltage (*V*_cell_) (Figure S21, Supporting Information) are used as predictor variables. The cycle aging case study's predictor variables are *ETP*, *T*, *SoC*_max_, *DoD* = *SoC*_max_ − *SoC*_min_, *P*_ch_, *EV*_ratio_, and time since superposed calendar aging effects are not subtracted from total aging.

#### 6.2.2 Stage One

The objectives of Stage One are to optimize hyperparameters for model creation and to generate a pool of promising models for seeding in Stage Two.

Thus, Stage One begins with optimizing 21 hyperparameters, which are considered as relevant for the performance and computational effort of the evolutionary process. These hyperparameters define general settings such as the population size and the number of generations, specify selection and reproduction mechanisms of the generational loop, and set boundaries for the structure of the models. For every hyperparameter, a range of allowed values is defined in Table S8, Supporting Information.

Since hyperparameters are interdependent and cannot be directly inferred from the available data, an additional algorithm is necessary for their optimization.^[^ ^18^ ^]^ In order to achieve an efficient and effective optimization of the relatively large amount of relevant hyperparameters, this framework applies Bayesian optimization via the *bayesopt* MATLAB function. As further explained in Note S1.2, Supporting Information, this algorithm iteratively varies the hyperparameters until a stop criterion is reached. During each iteration, one evolutionary run is performed on training data and evaluated on validation data. The resulting optimized settings are used for model creation.

The model creation process comprises 50 evolutionary runs to account for probabilistic variance in performance. These runs utilize training and validation data for learning (learning set) to compensate for the relatively small datasets usually available for lifetime prediction. This is possible since a separate validation set is no longer needed after completing hyperparameter optimization. The test set remains reserved for a‐posteriori performance evaluations. Each evolutionary run ends after the specified number of generations with the amount of models defined by the population size. For each of the 50 runs, three champion models are chosen. Stage One concludes by selecting 25 seeding models out of the resulting pool of 150 champion models. This selection process has a major impact on Stage Two and thus on the output of the framework.

#### 6.2.3 Stage Two

Stage Two builds on the results of the previous stage to further improve predictive accuracy and, most importantly, the reliability of the framework's output. For this, the processes of model creation and selection are repeated with the same learning set as used in Stage One. The differences are that the initial population of each evolution consists of the previously generated 25 seeding models instead of randomly generated ones and that different selection algorithms are used to focus on selecting one well generalizable model as final output, the apex model.

#### 6.2.4 Selection Mechanisms

At multiple stages of the algorithm, mechanisms must select suitable models from a large pool of potential solutions. In particular, these are "Champion Selection" for each evolutionary process, "Seeding/Apex Model Selection" at the end of each experiment of Stage One/Two, and "Experiment Selection" in order to finalize each stage. The main challenge is to avoid overfitting models even though model evaluation and selection are solely based on learning data since test data is reserved for a‐posteriori performance evaluations. Reliability and robustness of the selection process are key besides model accuracy and complexity. The accuracy of a model *j* is quantified by the root mean square error

(2) $$RMSE_{j}=\sqrt{\frac{1}{n}\sum_{i=1}^{n}{\left(y_{i}-{\hat{y}}_{i}\right)}^{2}}$$

with the predicted (${\hat{y}}_{i}$) and actual values (*y*_i_) of the response variable *y* for a dataset with *n* values. The *RMSE*'s unit depends on the investigated predictor variable. Predictions of *SoH*_C_ in percent (%) are evaluated by *RMSE* in percentage points (p.p.). For the evaluation of model complexity, expressional complexity is used. The expressional complexity of mathematical equations represented as tree structures is the sum of nodes of all possible subtrees.^[^ ^55^ ^]^

Champion Selection completes each evolutionary run of the model creation processes of Stage One and Stage Two by selecting three champion models. For this, each run's final population is filtered regarding basic requirements of robustness—as described in Note S1.3, Supporting Information—and ranked according to the multi‐objective fitness function

(3) $$\begin{array}{c} \begin{array}{c} Fitness_{1,j}=1-\sqrt{0.25\cdot {\left(1-\frac{RMSE_{\mathrm{learning},j}}{RMSE_{\mathrm{learning},\max}}\right)}^{2}+0.75\cdot {\left(1-\frac{Comp_{j}}{Comp_{\max}}\right)}^{2}} \end{array} \end{array}$$

with each model's error *RMSE*_learning_ on learning data and expressional complexity *Comp* set in relative terms to the corresponding maximum values in the population. To favor rather generalizable models, the complexity term is given more weight than the error term. Since each stage consists of 50 evolutionary runs, this process results in a pool of 150 champion models per stage.

Out of this pool of models, Seeding Model Selection identifies 25 seeding models in Stage One and Apex Model Selection chooses one apex model in Stage Two. For both selection processes, only the best 40% of champion models regarding *RMSE*_learning_ are considered. Even though the selection processes are optimized for each stage's goals, their structure is similar. Both assume that the majority of evolutionary runs concludes with reasonable champion models and that overfitting champion models are outliers. They enhance robustness by reliably eliminating these negative outliers with the drawback of potentially eliminating positive outliers as well. For this, the crowd error

(4) $$RMSE_{\mathrm{crowd},j}=\sqrt{\frac{1}{n}\sum_{i=1}^{n}{\left({\tilde{y}}_{i}-{\hat{y}}_{i}\right)}^{2}}$$

with the prediction median ${\tilde{y}}_{i}$ of all evaluated champion models is introduced as additional reference. While *RMSE*_crowd_ relies on test set input data for Stage One, it puts increased focus on generalizability for Stage Two by analyzing a more extensive artificial grid of various aging conditions. As described in Note S1.4, Supporting Information, the crowd error is relevant for adaptive thresholds, which models need to pass for further evaluation (see also Figures S22–S24, Supporting Information). In Stage One, the 25 best remaining models are selected for seeding according to their weighted relative error

(5) $$\begin{array}{ccc} WRE_{j} & = & \frac{2}{3}\frac{RMSE_{\mathrm{learning},j}}{RMSE_{\mathrm{learning},\max}-RMSE_{\mathrm{learning},\min}}\\ & & +\;\frac{1}{3}\frac{RMSE_{\mathrm{crowd},j}}{RMSE_{\mathrm{crowd},\max}-RMSE_{\mathrm{crowd},\min}} \end{array}$$

In contrast, Stage Two requires the selection of a single apex model. For this, the remaining models are evaluated by the multi‐objective fitness function

(6) $$\begin{array}{c} Fitness_{2,j}=\sqrt{0.5\cdot {\left(\frac{RMSE_{\mathrm{learning},j}}{RMSE_{\mathrm{learning},\max}}\right)}^{2}+0.5\cdot {\left(\frac{Comp_{j}}{Comp_{\max}}\right)}^{2}} \end{array}$$

which tolerates high errors and complexities less than *Fitness*_1_ (Figure S25, Supporting Information).

To attenuate the risk resulting from non‐deterministic algorithms, Stage One and Stage Two are evaluated in two independent experiments each. For both stages, Experiment Selection compares the *RMSE*_learning_ of each experiment's best‐ranked model according to *Fitness*_2_ and selects the experiment with the smaller error. In case of equal errors, lower model complexity is preferred.

## Conflict of Interest

The authors declare no conflict of interest.

## Author Contributions

K.S., J.S., S.H., and K.P.B. conceived and designed the project. K.S. and F.L. developed and evaluated the genetic programming framework with input from J.S. and S.H. K.S. and G.G. performed the benchmark analysis. J.S. and S.H. provided the ageing data. K.P.B. supervised the project. K.S. wrote the manuscript with contributions from F.L., J.S., S.H., and G.G. All authors discussed the results and reviewed the manuscript.

## Supporting information

Supporting Information

## Data Availability

The data that support the findings of this study are available from the corresponding author upon reasonable request.
