# Supplementary material for: Machine Learning‐Based Lifetime Prediction of Lithium‐Ion Cells
Source: Adv Sci (Weinh). 2022 Aug 26;9(29):2200630. doi: 10.1002/advs.202200630 (PMC9561774; doi:10.1002/advs.202200630)
Supplement: Supplementary file 1 — Supporting Information [file ADVS-9-2200630-s001.pdf]

Supporting Information

Machine Learning Based Lifetime Prediction of Lithium-Ion Cells

Kai Schofer [a,b*], *Florian Laufer* [a,b], *Jochen Stadler* [a,c], *Severin Hahn* [a], *Gerd Gaiselmann* [a], *Arnulf Latz* [c,d] *and Kai P. Birke* [b]

[*] Corresponding author: kai_axel.schofer@mercedes-benz.com

[a] Mercedes-Benz AG, Mercedesstraße 120, 70327 Stuttgart, Germany

[b] University of Stuttgart, Institute for Photovoltaics, Electrical Energy Storage Systems, Pfaffenwaldring 47, 70569 Stuttgart, Germany

[c] Ulm University, Institute of Electrochemistry, Albert-Einstein-Allee 47, 89081 Ulm, Germany

[d] Helmholtz Institute for Electrochemical Energy Storage (HIU), Helmholtzstraße 11, 89081 Ulm, Germany

The Supporting Information contains detailed explanations of:

Note S1: Machine Learning Framework

Note S1.1: Evolutionary Process

Note S1.2: Hyperparameter Optimization

Note S1.3: Robustness Filters for Champion Selection

Note S1.4: Adaptive Thresholds for Seeding / Apex Model Selection

Note S2: Benchmark Analysis

Note S2.1: (Semi-)Empirical Calendar Aging Models

Note S2.2: (Semi-)Empirical Cycle Aging Models

Note S2.3: Machine Learning Approaches

Note S3: Interpretability of Evolutionary Generated Models

Note S3.1: Model Structure

Note S3.2: Integration of Domain Knowledge

This is followed by:

Supporting Figures

Supporting Tables

Supporting References

Note S1: Machine Learning Framework

Note S1.1: Evolutionary Process

The core of our framework relies on an adapted version of the GPTIPS 2 framework for multi-gene symbolic regression via genetic programming developed by Searson et al.[1]. The GPTIPS 2 framework was chosen because it is open source, optimized for multi-gene symbolic regression, well established, highly customizable and programmed in MATLAB, which is commonly used in vehicle development.
Its evolutionary process, which is summarized in Figure S5 (Supporting Information), starts with the creation and evaluation of an initial population. This is followed by a generational loop, which is iterated until a termination criterion is satisfied and the final generation's best individuals are returned. The generational loop consists of parent selection, reproduction, fitness evaluation and survivor selection.[2–5]

While **initialization** originally relies exclusively on the random generation of trees,[1] we added the opportunity to seed variable proportions of models, which are e.g. domain knowledge based. Seeding improves the initial population's fitness and hence potentially increases the algorithm's efficiency. Additionally, it can direct the optimization process towards regions of the search space containing good solutions and thereby enhance its effectiveness. However, also the opposite effect is possible resulting from narrowing the search space. The influence of seeding on the algorithm's performance depends on the investigated problem.[2,4,6] Furthermore, the adapted version only utilizes the ramped half and half method for the random generation of trees due to its advantages regarding diversity in comparison to the other tree creation methods available in GPTIPS 2.[1,4]

For **fitness evaluation**, the gene weighting coefficients of the individuals are estimated by least squares regression. In GPTIPS 2 the fitness values are determined either under sole consideration of the *RMSE* on training/learning data (single-objective) or by non-dominated sorting (multi-objective).[1] Since the single-objective fitness function does not take into account the multi-objective task of predictive modelling, it is replaced by the scaled multi-objective fitness function $Fitness_1$ in the modified version of the algorithm. This approach transfers multiple objectives into a single-objective problem by weighting the objective specific evaluation functions for model error (*RMSE* on training/learning data) and model complexity (expressional complexity). This is implemented to offer a computationally more efficient option

for multi-objective fitness determination than non-dominated sorting. However, in comparison to non-dominated sorting, the assumption of structure and weights of the combined fitness function can be disadvantageous as it reduces the area of interest to a line of pre-defined form before the range of possible solutions is known (Figure S25, Supporting Information).[3,4,7]
The non-dominated sorting algorithm takes into account the same inputs as the multi-objective fitness function: accuracy measured by the *RMSE* on training/learning data and complexity quantified either by expressional complexity or by the simpler approach of node counting, which is not considered by the modified version of the algorithm.[1] An individual is not dominated if it achieves the best fitness regarding one objective compared to all individuals of the population that share its fitness level regarding the other objective. If an individual is not dominated by any feasible solution, it is Pareto-optimal. In non-dominated sorting all individuals are assigned to a non-domination rank based on the worst ranked individual they are dominated by.[2–4] In GPTIPS 2, individuals of the same non-domination rank are sorted randomly (secondary sorting).[1]

For **parent selection,** only the tournament method is supported. Tournament selection is a computationally efficient solution for parent selection because it does not require knowledge of the entire population. Each parent is selected by an independent tournament for which a defined number of individuals is randomly picked from the population. Out of these individuals, the one with the best fitness is selected. Depending on probabilistic choice, each tournament relies on one of the supported fitness evaluation methods.[1,4]

Reproduction creates new individuals from previously selected parents in order to explore the space of possible solutions. In genetic programming, the genetic operators recombination, manipulation and cloning are applied mutually exclusive depending on probabilistic choice.[2,4]
Recombination creates offspring, which contain a combination of the genetic material of their parents. The recombination techniques offered by GPTIPS 2 are subtree crossover (low level) as well as gene crossover (high level).[1,2,4]
The purpose of manipulation is the introduction of new genetic material by modification of existing individuals.[2,4] For manipulation, subtree mutation and five types of point mutation are available. The point mutation techniques either exchange non-constant terminals (type 1) or modify constant terminals by adding values based on Gaussian distributions (type 2), by

substituting them with randomly generated values (type 3), by setting them to zero (type 4) or by setting them to one (type 5).[1]

While the other genetic operators modify genetic material to explore the solution space, cloning preserves genetic material to avoid a decline in fitness. Each offspring created by cloning is an exact copy of its parent.[2]

The choice of a specific genetic operator is probabilistic. In GPTIPS 2 the corresponding probabilities remain constant throughout the evolutionary process.[1] However, the efficiency of an evolutionary algorithm can be improved by adjusting the probability of mutation to local solution space characteristics.[3] Therefore, we introduced an adaptive rate of mutation according to Rechenberg's 1/5 success rule. The modified algorithm adapts the mutation rate starting with the eleventh generation of each evolutionary process. For this, the previous ten generations are compared regarding their $RMSE_{\mathrm{min}}$ on training/learning data. In case of more than two improvements during these generations, the probability for mutation is increased by an adaptation rate of 0.1. Correspondingly, the probability for mutation is decreased in case of less than two improvements. Compliance with the requirements of the evolutionary algorithm is ensured by scaling of all other reproduction related probabilities as well as by enforcing constraints.

The **survivor selection** of GPTIPS 2 relies on the replace-worst method. This allows for the survival of the fittest previously existent individuals. Even though this enables quick improvements of the population's fitness, it has to be considered that it can also lead to premature convergence.[1,4]

Possible **termination criteria** are the number of generations, the computational time and the best individual's fitness.[1]

While GPTIPS 2 selects the individual, which is returned as best solution, from all individuals of the final generation based on their *RMSE* on training/learning data,[1] the modified algorithm utilizes the selection mechanism described in Methods for champion selection during model creation and the selection mechanism described in *Note S1.2: Hyperparameter Optimization* (Supporting Information) for champion selection during hyperparameter optimization.

Note S1.2: Hyperparameter Optimization

Hyperparameters tune the behavior of learning algorithms.[8] The 21 hyperparameters summarized in Table S8 (Supporting Information) are considered for optimization due to their suspected relevance to the performance of the evolutionary algorithm utilized in our framework. Our framework optimizes these hyperparameters via the MATLAB function *bayesopt* for Bayesian optimization. Further hyperparameters of the evolutionary algorithm are not optimized as they are expected either to have no significant influence on its performance or to require further research. Therefore, they are not changed from their default settings. More details can be found in the documentation for GPTIPS 2, cf. [1].

The Bayesian optimization algorithm iteratively performs runs of the evolutionary algorithm on training data with varying settings. For this, the allowed values for each hyperparameter are defined by minimum, maximum and step size. In addition, constraint functions ensure that all hyperparameter variations meet the requirements of the evolutionary algorithm.

During the first five iterations, the hyperparameters are selected randomly in order to build an initial surrogate model. After each subsequent iteration, this Gaussian process surrogate model is updated by fitting it to all previous observations. Based on this probabilistic model, an acquisition function selects the next point for evaluation, cf. [9]. To reliably find good global solutions, which are also computationally efficient, the acquisition function *expected improvement - per second - plus* is chosen in combination with an exploration ratio of 0.6.

For each iteration of the optimization algorithm, the final population of the corresponding evolutionary process is evaluated on validation data. The individual passing a filter regarding robustness (see *Note S1.3: Robustness Filters for Champion Selection,* Supporting Information) and achieving the minimum $RMSE_{\text{validation}}$ is selected as champion. The quality of an iteration is assessed by the predictive error of its champion model quantified by

$$1 - R^2 = 1 - \frac{\sum_{i=1}^{n}(y_{\mathrm{i}} - \hat{y}_{\mathrm{i}})^2}{\sum_{i=1}^{n}(y_{\mathrm{i}} - \bar{y})^2} \tag{S1}$$

with the predicted values $\hat{y}_{\mathrm{i}}$, the actual values y_{i} and the mean of the actual values $\bar{y}$ of the response variable y for validation datasets with n values. It is taken into account that the evolutionary algorithm's results are stochastic.

The optimization algorithm is executed until the champion of an evolutionary process with a maximum execution time of 30 min achieves a predictive error of $1 – R^2 < 0.01$. To avoid unnecessary iterations, the minimization target $1 – R^2$ is gradually increased by 0.01, whenever

the preceding 100 iterations did not enhance accuracy. Further termination criteria are the maximum amount of iterations n_{max} = 500 and the maximum simulation time t_{max} = 72 h.

Note S1.3: Robustness Filters for Champion Selection

During hyperparameter optimization, the selection of champion models, which only perform well on validation data, is avoided by limiting further evaluation to models, which are non-dominated on training data. The remaining individuals are compared by their $RMSE_{training}$ on training data. Only the best 40 % of models are considered for champion selection as described in *Note S1.2: Hyperparameter Optimization* (Supporting Information).

For champion selection **during model creation**, first a pseudo test set is generated by linearly extrapolating the predictor variables over storage time or energy throughput and by predicting the response variable for these inputs with a state-of-the-art cell aging model. The coefficients of this model are estimated on the available learning data with the MATLAB function *fitnlm* for non-linear regression. Model structure and start values of the coefficients are obtained from the connected cell aging database. The resulting pseudo test set starts at begin of life and covers five times the storage time or energy throughput of the learning data. Models are excluded from further evaluation if

- their predictions for the pseudo input data include increasing SoH_C values or $SoH_C = \pm\infty$,
- their normalized mean absolute error $NMAE = \frac{1}{n}\frac{\sum_{i=1}^{n}|y_i-\hat{y}_i|}{y_{max}-y_{min}} \geq 0.3$ for learning set or pseudo test set with the predicted values $\hat{y}_i$, the actual values y_i, the minimum and maximum values $y_{min/max}$ of the response variable y for datasets with n values,
- they are dominated on learning data.

If no individual meets these basic requirements of robustness, the evolutionary run is restarted. Otherwise, the remaining individuals are compared by their $RMSE_{learning}$ on learning data. Only the best 25 % of models are considered for champion selection as described in Methods.

Note S1.4: Adaptive Thresholds for Seeding / Apex Model Selection

The reliable selection of generalizable models during and after model creation of *Stage One* and *Stage Two* is particularly challenging since

- the information of training and validation set is already used during hyperparameter optimization and thus is known to the models,
- the test set is reserved for a-posteriori performance evaluations,
- the relatively small sample sizes usually available for lifetime prediction of lithium-ion cells discourage the additional hold-out of data sets for model selection.

In order to enable the selection of generalizable instead of overfitting models, this unavailability of unknown data needs to be compensated. For this, the concept of the crowd error $RMSE_{\mathrm{crowd}}$ is introduced. It assumes that the majority of evolutionary runs concludes with reasonable champion models and that overfitting champion models are outliers. The mechanism calculates the median $\tilde{y}$ of the predictions of all eligible models for the input data of test sets (*Stage One*) or artificially generated grids (*Stage Two*). As shown in Figure S22, $\tilde{y}$ resembles the trend of the test data's response variable despite not using this information. Therefore, the comparison of a single model's predictions with $\tilde{y}$ in form of $RMSE_{\mathrm{crowd}}$ can be used as indication of a model's likeliness for overfitting while unknown data is not available. The crowd error is relevant for adaptive thresholds, which models need to pass for further evaluation.

In *Stage One*, models need to meet the requirement

$$RMSE_{\mathrm{crowd,j}} \leq 5 * \widetilde{RMSE}_{\mathrm{learning}} \tag{S2}$$

with the median $\widetilde{RMSE}_{\mathrm{learning}}$ of all eligible models. This threshold filters out overfitting models since they contradict the trend of $RMSE_{\mathrm{test}}$, which is approximated by $RMSE_{\mathrm{crowd}}$, decreasing with $RMSE_{\mathrm{learning}}$.

To put increased focus on generalizability in *Stage Two*, the learning data's aging conditions are extended to artificial grids as illustrated in Figure S23. For calendar aging, the grid covers three evenly distributed temperatures and five evenly distributed *SoC*s limited by each factor's minimum and maximum values available in the learning data. For cycle aging, the grid comprises all available factor levels for temperature and - after the exclusion of minimum and maximum - all available levels of the remaining stress factors. These conditions are extrapolated over twice the maximum storage time or energy throughput observable in the learning data.

All eligible models *j* are evaluated by $RMSE_{\text{crowd,j,a}}$ and its standard deviation $\sigma_{\text{crowd,a}}$ individually for each aging condition *a* of the artificial grids. A generalizable model is expected to perform well for various aging conditions. Thus, models have to comply with the requirement

$$RMSE_{\text{crowd,j,a}} \leq RMSE_{\text{crowd,min,a}} + x_{\text{thresh}} * \sigma_{\text{crowd,a}} \tag{S3}$$

for all aging conditions *a* of the artificial grid. Initially, the scaling factor x_{tresh} is assigned a value of 0.5 to limit the number of models passing the threshold. However, as long as less than 10 models satisfy Equation S3 (Supporting Information), x_{tresh} is iteratively increased by 0.5.

The stepwise progression of the selection mechanism is exemplarily shown in Figure S24a (Supporting Information) for *Stage One* and Figure S24b (Supporting Information) for *Stage Two* of test case *Cal Ext*$_{20}$*/Int*$_{2}$.

Note S2: Benchmark Analysis

Due to the large variety of aging models and their dependency on the investigated stress factors, we refrain from a comprehensive review of aging models. Instead, only models, which can be applied to our aging data, are considered as benchmark.

Note S2.1: (Semi-)Empirical Calendar Aging Models

Recent advances in modelling calendar aging were made by Hahn et al.[10] and Schimpe et al.[11]. The model proposed by Hahn et al. considers an initial loss of lithium during formation, the one by Schimpe et al. improves *SoC* dependency by implementing an anode potential dependency. These approaches require additional information obtained by specific begin of life experiments, cf. [10,11]. However, to ensure a fair comparison between all investigated approaches, all models are required to use the same data. Therefore, these models are excluded from further evaluation.

As a result, the benchmark analysis of semi-empirical calendar aging models is limited to the models summarized in Table S1 (Supporting Information). These models represent the most common approaches for predicting calendar aging with a semi-empirical model. Their fitting parameters p_i are determined by the MATLAB function *lsqcurvefit* for least squares regression. All investigated models show similar predictive performance in the calendar aging case study (Figure S13, Supporting Information). Of the relevant models, t^{p_4} achieves the lowest predictive error $\overline{RMSE}$ averaged over all test cases (Table S1, Supporting Information). Therefore, it is selected to represent (semi-)empirical approaches for calendar aging. In Table S6 (Supporting Information), the predictive errors $RMSE_{test}$ of the t^{p_4} model are compared with each test case's apex model of the genetic programming framework.

Note S2.2: (Semi-)Empirical Cycle Aging Models

The higher complexity of cycle aging leads to a large diversity in investigated stress factors and resulting cycle aging models.[12–15] Therefore, most models are designed for a specific dataset and cannot be applied to other datasets.

As a result, only the models summarized in Table S2 (Supporting Information) are applicable to the data utilized in this work. The fitting parameters p_i are obtained by least squares regression (MATLAB function: *lsqcurvefit*) for ETP^{p_2} and by stepwise regression (MATLAB func-

tion: *stepwiselm*) for ETP_{Regr}. The ETP^{p_2} model is incapable of creating a global fit and requires an individual fit per aging condition instead. Therefore, this modelling approach cannot make aging predictions over cell aging conditions. In contrast, ETP_{Regr} is able to perform all common tasks of lifetime prediction (Figure S14, Supporting Information). As a result, ETP_{Regr} is selected to represent (semi-)empirical approaches for cycle aging. In Table S7 (Supporting Information) the predictive errors $RMSE_{\mathrm{test}}$ of the ETP_{Regr} model are compared with each test case's apex model of the genetic programming framework.

Note S2.3: Machine Learning Approaches

The machine learning approaches selected for the benchmark analysis are investigated in Python. For this, the software packages summarized in Table S3 (Supporting Information) are used.

In order to tune their hyperparameters comparable to our genetic programming framework, the Tree of Parzen algorithm implemented in the *hyperopt* python package is applied, cf. [16]. Exceptions are Gaussian process regression and fast function extraction: For Gaussian process regression, the interval hyperparameter tuning module available in the used python package is applied. Furthermore, no hyperparameters of the fast function extraction algorithm are tuned, as its authors claim that their algorithm is hyperparameter insensitive (cf. [17]).

Table S3 (Supporting Information) gives an overview of the optimized parameters (all other parameters are set to the standard setting of each package) and the parameter search spaces. The network architecture of the multi-layer perceptron is defined by two hidden layers with rectified linear unit (ReLU) activation functions and dropout rates per hidden layer.

Analysis of the performance of all investigated algorithms regarding calendar and cycle aging prediction (Figure S15, Supporting Information) reveals that the different types of machine learning greatly differ in their strengths and weaknesses. However, of all investigated approaches, extreme gradient boosting achieves the lowest predictive error $\overline{RMSE}$ averaged over all test cases (Table S3, Supporting Information). Therefore, it is selected to represent established machine learning approaches in the case study. In Table S6 (Supporting Information) and Table S7 (Supporting Information), the predictive errors $RMSE_{\mathrm{test}}$ of extreme gradient boosting are compared with each test case's apex model of the genetic programming framework.

Note S3: Interpretability of Evolutionary Generated Models

Note S3.1: Model Structure

For a concise overview, the apex models of the test cases *Cal Ext*$_{20}$, *Cal Int*$_{1}$, *Cal Ext*$_{SoC}$, *Cyc Ext*$_{80}$, *Cyc Int*$_{DoD}$ and *Cyc Ext*$_{T}$ – and thus examples for every relevant task of lifetime prediction – are shown.

***Cal Ext*$_{20}$**

$$SoH_\mathrm{C} = 102.1 - 3318.0\sqrt{t} - 3.805\sqrt{t}\sqrt{T\sqrt{V_\mathrm{cell}}\,(T + 39.64)} + 182.7\sqrt{t}\sqrt{T + V_\mathrm{cell} + 213.2} + 25.42\sqrt{t}\sqrt{T\,V_\mathrm{cell}} + 0.258\frac{\sqrt{2\,t + 5.458}}{V_\mathrm{cell} - 4.2988}$$

***Cal Int*$_{1}$**

$$SoH_\mathrm{C} = 101.6 - 1031.0\sqrt{t} - 3.31\sqrt{t}\left(T + V_\mathrm{cell} + \sqrt{e^{(1.944\cdot 10^{-8})\,V_\mathrm{cell}{}^{3\,V_\mathrm{cell}}}}\right) + 117.6\sqrt{t}\sqrt{\left(T + 3.359\cdot 10^{-3}\,V_\mathrm{cell}{}^{V_\mathrm{cell}}\right)}$$

***Cal Ext*$_{SoC}$**

$$SoH_\mathrm{C} = 101.4 - 2.902\cdot 10^{-3}\sqrt{t}\sqrt{T\,e^{\sqrt{T}}} - 7.625\cdot 10^{-4}\sqrt{t}\,T^2 + 6.202\cdot 10^{-2}\sqrt{t}\sqrt{e^{\sqrt{T}}} - 2.083\cdot 10^{-7}\sqrt{t}\,T\,ln(V_\mathrm{cell})^{0.08994\,V_\mathrm{cell}{}^4}$$

***Cyc Ext*$_{80}$**

$$SoH_\mathrm{C} = 100.5 - 5.689\cdot 10^{-3}\,T - 2.845\cdot 10^{-3}\sqrt{ETP}\,EV_\mathrm{ratio} - 2.328\cdot 10^{-6}\left(\frac{DoD\,P_\mathrm{ch}}{T}\right)^2 + 7.898\cdot 10^{-2}\left(\frac{P_\mathrm{ch}}{T} + \sqrt{SoC_\mathrm{max}}\right) + 1.286\cdot 10^{-1}\sqrt{ETP} + 3.949\cdot 10^{-2}\,(DoD - SoC_\mathrm{max}) + 5.335\cdot 10^{-6}\,t\,SoC_\mathrm{max}\,(DoD - SoC_\mathrm{max})$$

***Cyc Int*$_{DoD}$**

$$SoH_\mathrm{C} = 105.0 - 7.063\cdot 10^{-5}\,ETP\,SoC_\mathrm{max} - 7.571\cdot 10^{-2}\,SoC_\mathrm{max} - 7.156\cdot 10^{-6}\,(T - 20.44)^2\,(t - P_\mathrm{ch}) - 1.936\cdot 10^{-3}\sqrt{ETP\,EV_\mathrm{ratio}{}^2} + 2.016\cdot 10^{-3}\,T\,(DoD - T)$$

***Cyc Ext*$_{T}$**

$$SoH_\mathrm{C} = 99.18 - 2.041\cdot 10^{-2}\,SoC_\mathrm{max} - 2.506\cdot 10^{-7}\sqrt{ETP}\,SoC_\mathrm{max}{}^2\,EV_\mathrm{ratio} - 2.709\cdot 10^{-5}\left(\frac{T\,SoC_\mathrm{max}}{DoD\,ln\left(\frac{T\,P_\mathrm{ch}\,SoC_\mathrm{max}\,EV_\mathrm{ratio}}{t}\right)}\right)^6 + 4.083\cdot 10^{-2}\,DoD + 6.804\cdot 10^{-3}\left(T - ETP - \frac{P_\mathrm{ch}}{T} + \sqrt{\frac{DoD}{|ln(T) - 0.1125\,T|}}\right)$$

Note S3.2: Integration of Domain Knowledge

As a first step towards the integration of domain knowledge, the seeding of semi-empirical models in initial populations of *Stage One* is investigated: The semi-empirical benchmark model t^{p_4} is seeded with proportions of 20 %, 40 %, 60 %, 80 % and 100 %. Furthermore, all four models of Table S1 (Supporting Information) and the extended t^{p_4} model

$$SoH_{\mathrm{C}} = 100 - p_1 \cdot A \cdot \left[\left(\frac{Liloss_{\mathrm{form.}}}{p_1 \cdot A} \right)^{\frac{1}{p_4}} + t \right]^{p_4} + Liloss_{\mathrm{form.}}$$

by Hahn et al.[10] with $A = e^{\frac{p_2}{T}} \cdot e^{p_3 \cdot V_{\mathrm{cell}}}$ and the lithium lost during formation $Liloss_{\mathrm{form.}}$ are seeded together with a proportion of 20 % each. To enable a detailed evaluation of the influence of seeding on the evolutionary process, this investigation is conducted with an adjusted version of the genetic programming framework with simplified seeding/apex model selection mechanisms:

- The 25 best ranked champion models of *Stage One* according to $Fitness_1$ are selected as seeding models.
- The best ranked champion model of *Stage Two* according to $Fitness_2$ is selected as apex model.

By replacing the complex selection mechanisms with these simple rankings, this investigation allows for conclusions regarding the influence of seeding on the evolutionary process with minimal interference from the selection mechanisms.

In Figure S20 (Supporting Information), the results of seeding are compared to random initialization of the initial populations of *Stage One*. On learning data, similar performances are achieved by all examined approaches. Seeding the t^{p_4} model with proportions of 20 % and 40 % even results in an increased accuracy on the learning set for the apex models of all investigated test cases ($Cal\ Ext_{20}$, $Cal\ Ext_{50}$, $Cal\ Ext_{65}$ and $Cal\ Int_1$). Also on test data, the apex models of some seeding approaches are of a similar accuracy as the apex models resulting from random initialization. However, on the test set, no approach of integrating domain knowledge consistently improves apex model accuracy across all investigated test cases. More importantly, seeding semi-empirical models enlarges the deviation of median, 75th and 25th percentile from the maximum accuracy. This drastically increases the risk of selecting an inferior model as apex model. These disadvantageous effects can be explained by a disturbance of the evolution caused by the incorporation of domain knowledge. Seeding semi-empirical models confines the search space too rapidly to local optima. This leads to excellent performance on data used for the generational loop but can result in models, which are not

properly generalizable. Nonetheless, integrating domain knowledge shows the potential of improving model accuracy. Therefore, further research should be conducted on reducing the large deviation of accuracy between champion models.

Furthermore, this investigation highlights the opportunity of enhancing existent theories with our algorithm as the apex models resulting from seeding resemble the seeded semi-empirical models. This is exemplarily shown for *Cal Ext*$_{65}$.

Structure of the semi-empirical model t^{p_4}

$SoH_C =$

$100 \; - p_1 \cdot$	Offset and fitting parameter for the term of stress factors
$e^{\frac{p_2}{T}} \cdot$	Temperature dependency
$e^{p_3 \cdot V_{\mathrm{cell}}} \cdot$	*SoC* / Voltage dependency
t^{p_4}	Time dependency

Apex model *Cal Ext*$_{65}$: 20 % seeding of t^{p_4}

$$SoH_C = 112.1 - 7.319 \cdot 10^7 \; e^{\frac{-10722.0}{T}} \; e^{2.873\,V_{\mathrm{cell}}} \; t^{0.5} \; \sqrt{T} - 3.705 \cdot 10^3 \frac{t + 330.1}{T^2} - 1.384 \cdot 10^5 \frac{t}{(V_{\mathrm{cell}} - 3.557)\,(2.0\,T + t + V_{\mathrm{cell}})^3} + 2.088 \cdot 10^{-4} \frac{t}{(V_{\mathrm{cell}} - 3.423)\,(V_{\mathrm{cell}} - 3.796)} + 0.5101 \frac{t - 7.808}{1.801\left(T - {V_{\mathrm{cell}}}^{V_{\mathrm{cell}}}\right) + 15.08}$$

Apex model *Cal Ext*$_{65}$: 40 % seeding of t^{p_4}

$$SoH_C = 112.2 - 4.668 \cdot 10^6 \; e^{\frac{-10666.0}{T}} \; e^{2.065\,V_{\mathrm{cell}}} \; t^{0.5} \; \sqrt{T^3} - 3.093 \cdot 10^3 \frac{t + 399.5}{T^2} - 0.0997 \frac{t}{(t + 203.6)\,(V_{\mathrm{cell}} - 3.559)} - 0.114 \frac{t}{T\,(V_{\mathrm{cell}} - 3.411)} - 0.110 \frac{t}{\left(T + 26.07 - {V_{\mathrm{cell}}}^{V_{\mathrm{cell}}}\right)}$$

Apex model *Cal Ext*$_{65}$: 60 % seeding of t^{p_4}

$$SoH_{\mathrm{C}} = 8039.0 - 2.57 \cdot 10^{5} \quad e^{\frac{-10499.0}{T}} \quad e^{4.739\,V_{\mathrm{cell}}} \quad t^{0.5929} - 1.189 \cdot 10^{4} \frac{t^{0.5929}}{5.498\,T^{2} - 4.942 \cdot 10^{5}} - 1.2522 \cdot 10^{4}\, e^{\frac{t}{e^{\sqrt{V_{\mathrm{cell}}}^{V_{\mathrm{cell}}}} + T^{2} + V_{\mathrm{cell}} - 35088.0}} + 4.582 \cdot 10^{3} e^{\frac{t}{e^{\sqrt{V_{\mathrm{cell}}}^{V_{\mathrm{cell}}}} + 69.74\,T + 3\,t}}$$

Apex model *Cal Ext*$_{65}$: 80 % seeding of t^{p_4}

$$SoH_{\mathrm{C}} = 100.4 - 1.857 \cdot 10^{5} \quad e^{\frac{-7397.0}{T}} \quad e^{2.48\,V_{\mathrm{cell}}} \quad t^{0.7512} - 32.76\,t + 5.023\,t\,V_{\mathrm{cell}} + 55.17 \frac{t}{V_{\mathrm{cell}}} + 8.619 \frac{t}{1.126\,ln(T) - 0.6179\,T\,V_{\mathrm{cell}}\,e^{-V_{\mathrm{cell}}}}$$

Apex model *Cal Ext*$_{65}$: 100 % seeding of t^{p_4}

$$SoH_{\mathrm{C}} = -16899.0 - 81.18 \quad e^{\frac{-12233.0}{T+4.933}} \quad e^{4.248\,e^{\sqrt{V_{\mathrm{cell}}}}} \quad t^{0.5725} + 51.0 \quad e^{\frac{-1620.0}{T}} \quad e^{e^{-\frac{(V_{\mathrm{cell}}+10.55)}{t}} - 153577\,e^{-V_{\mathrm{cell}}^{2}}} \quad t^{0.5091} \quad (V_{\mathrm{cell}} - 4.576) - 4.98\,T + 7737.0 \sqrt{ln\left(T^{e^{-\frac{T}{e^{2\,t} + t^{3} + (T+t-6.956)^{3}}}}\right)}$$

Apex model *Cal Ext*$_{65}$: Seeding of five semi-empirical models (20 % each)

$$SoH_{\mathrm{C}} = 86.66 - 1.146 \cdot 10^{7} \quad e^{\frac{-16566.0}{T}} \quad e^{8.164\,V_{\mathrm{cell}}} \quad t^{0.6698} + 4.596 \cdot 10^{3} \frac{6.062^{T^{3} - V_{\mathrm{cell}}}}{1.1\,T + 2.199\,t}$$

Supporting Figures

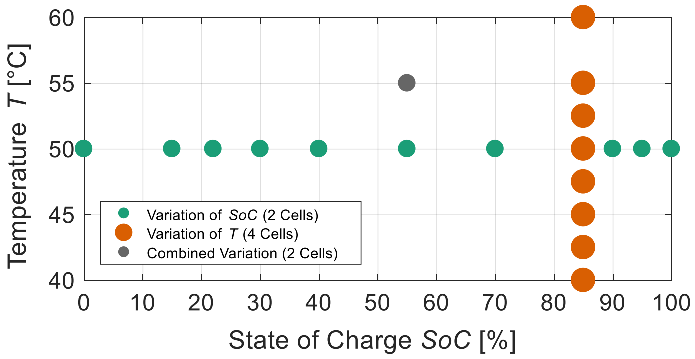

Figure S1. Design of experiment matrix for calendar cell aging: Temperature *T* and state of charge *SoC* were varied, while the effective storage time remained constant. Each storage condition was investigated with multiple cells.

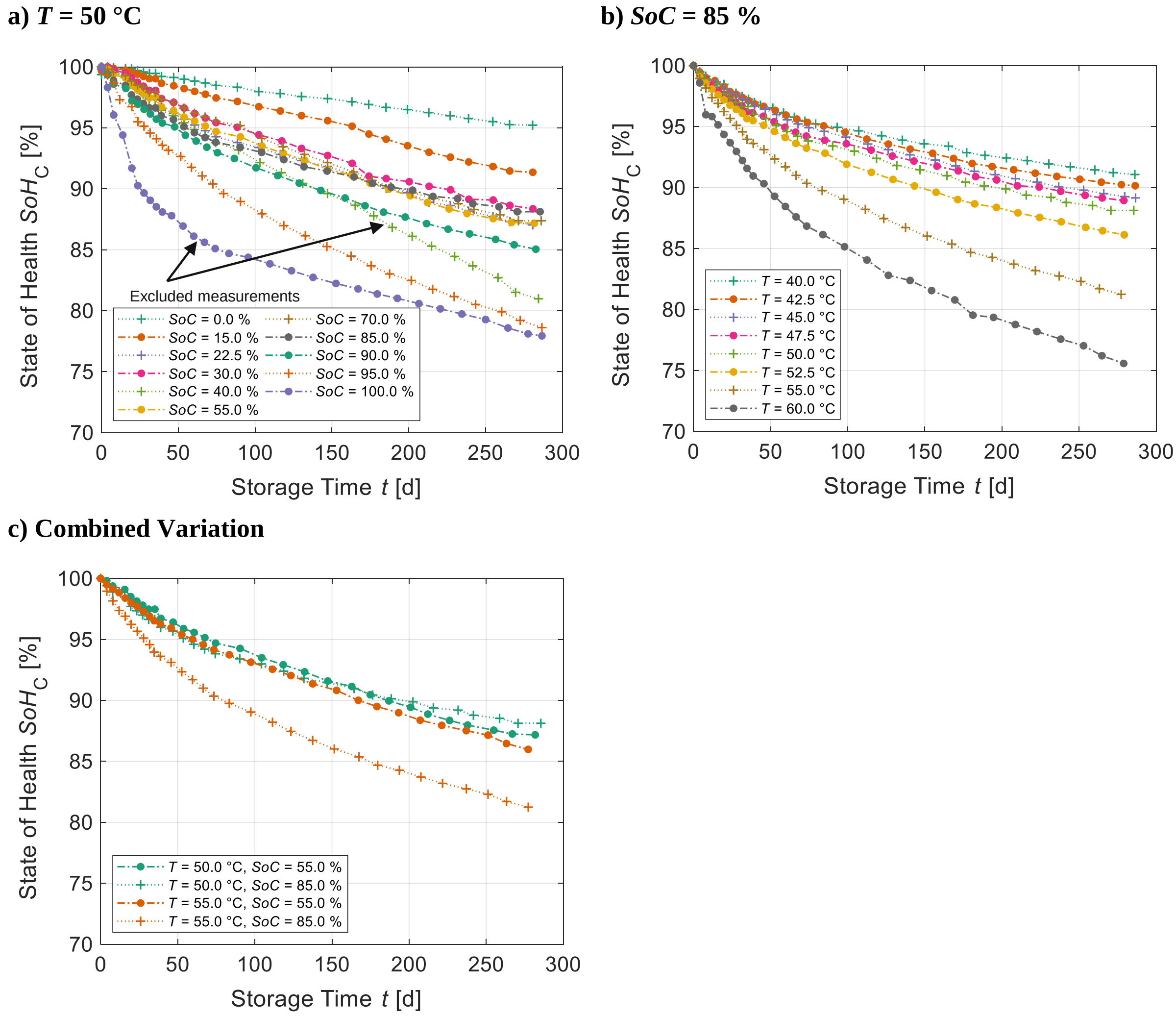

Figure S2. Evolution of mean SoH_C over t for the calendar aging experiment: Influence of **a)** *SoC* and **b)** *T* on aging behavior. **c)** Interdependent influence of *SoC* and *T*. The results of cells aged under the same conditions are averaged to avoid prioritization of conditions investigated with more cells and to reduce the influence of minor fluctuations in measured capacity on the modelling process. As a result, each of the 19 investigated storage conditions is represented by one test series. For more clarity, standard deviations are not shown. The cells aged at 40 % and 100 % *SoC* at *T* = 50 °C are excluded due to their abnormal aging behavior.

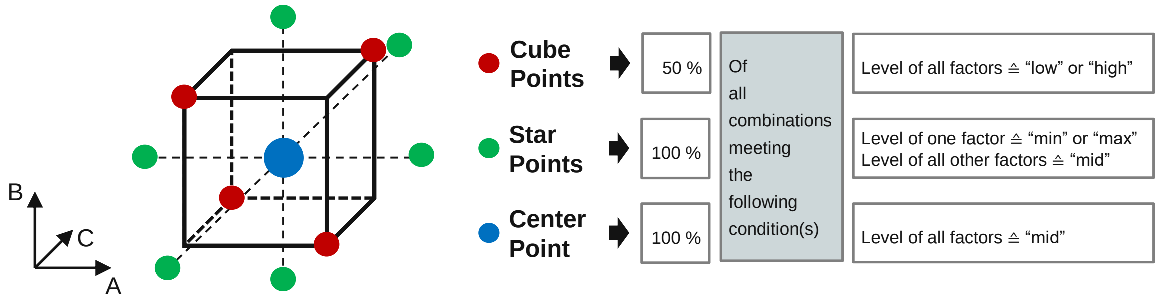

Figure S3. Exemplary scheme of the design of experiment for cycle cell aging: Central composite design for three factors (A, B and C). It consists of a two-level half fractional factorial design (cube points) extended by a center point and star points. Each set of points comprises combinations of factor levels meeting specific conditions.

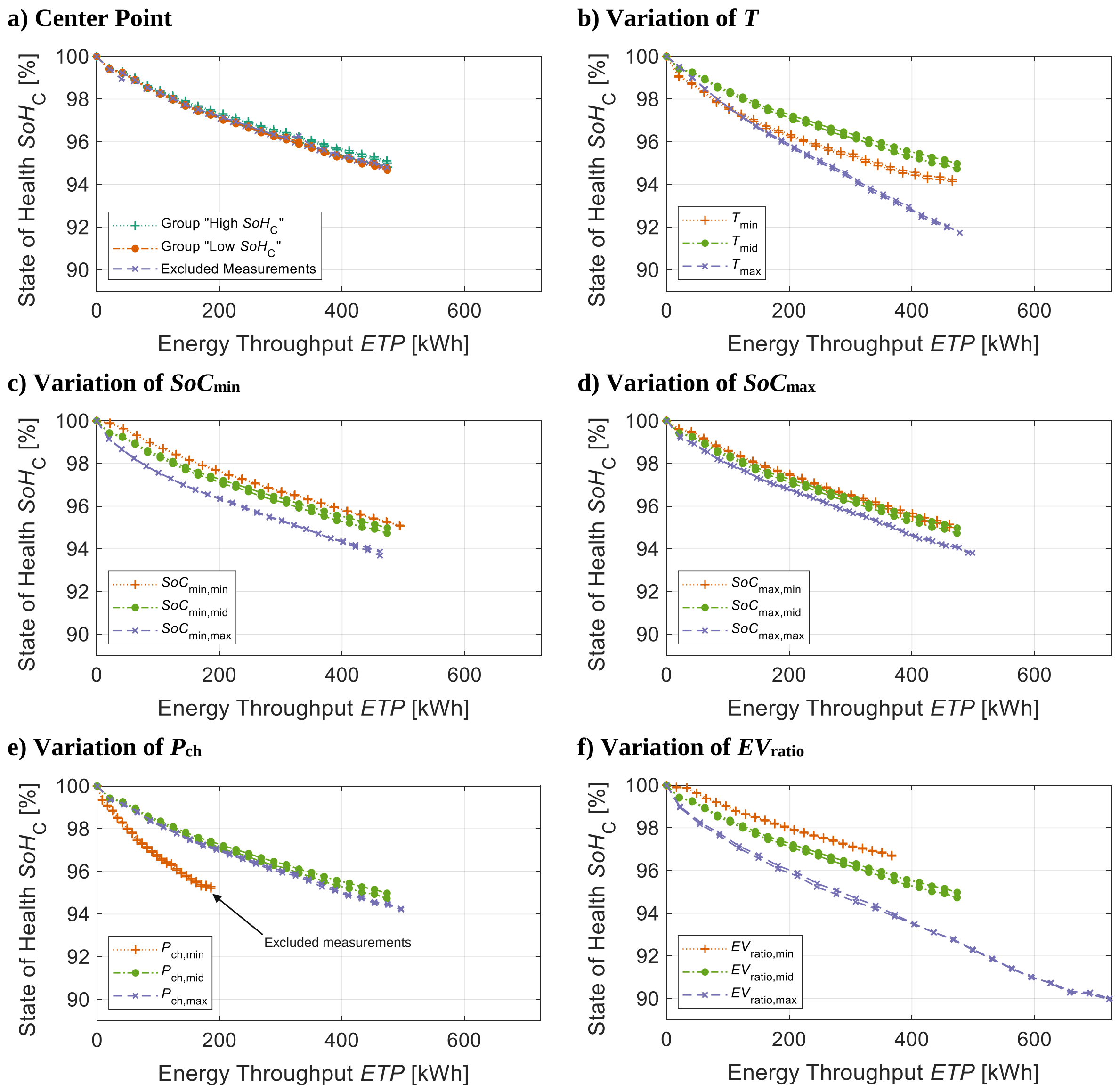

Figure S4. Evolution of *SoH*C over *ETP* for the cycle aging experiment: a) To avoid prioritization of the center point, its tests are sorted into two equally sized groups ("High SoH_C" and "Low SoH_C" based on the SoH_C at the end of each test) and the measurement results are averaged for both groups. Two tests are excluded due to unrepresentative deviations in SoH_C. In subsequent figures, the average of each group is shown without standard deviations. Consequently, each of the 27 investigated operating conditions is represented by two test series. The usage of two test series per condition instead of one allows for an examination of the capability to deal with fuzziness during the modelling process. Isolated influence of **b)** T, **c)** SoC_{min}, **d)** SoC_{max}, **e)** P_{ch} and **f)** EV_{ratio}. For this, the center point is compared with star points. The star point, for which P_{ch} was set to "min", is excluded because it primarily investigated calendar cell aging.

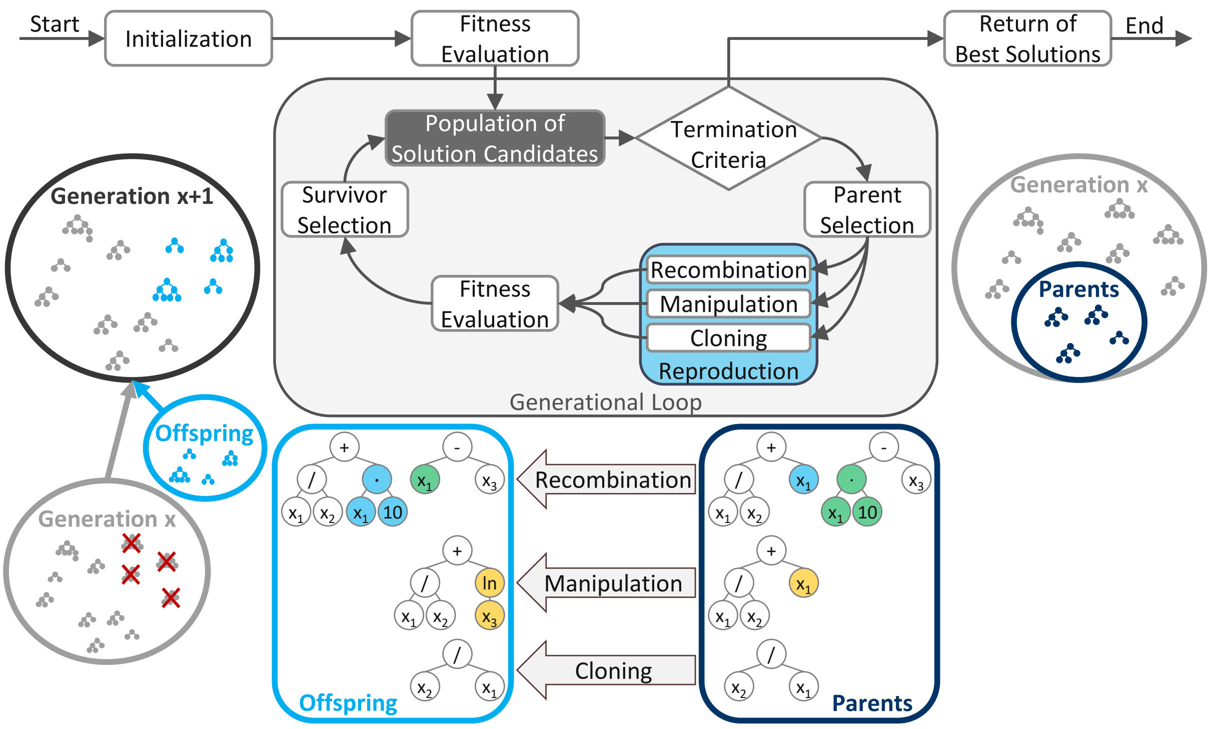

Figure S5. Evolutionary process of the genetic programming framework: Following the creation and evaluation of an initial population, a generational loop is iterated until a termination criterion is satisfied and the final generation's best individuals are returned. Variation by reproduction combined with fitness based selection drives this optimization process. Based on [2–5].

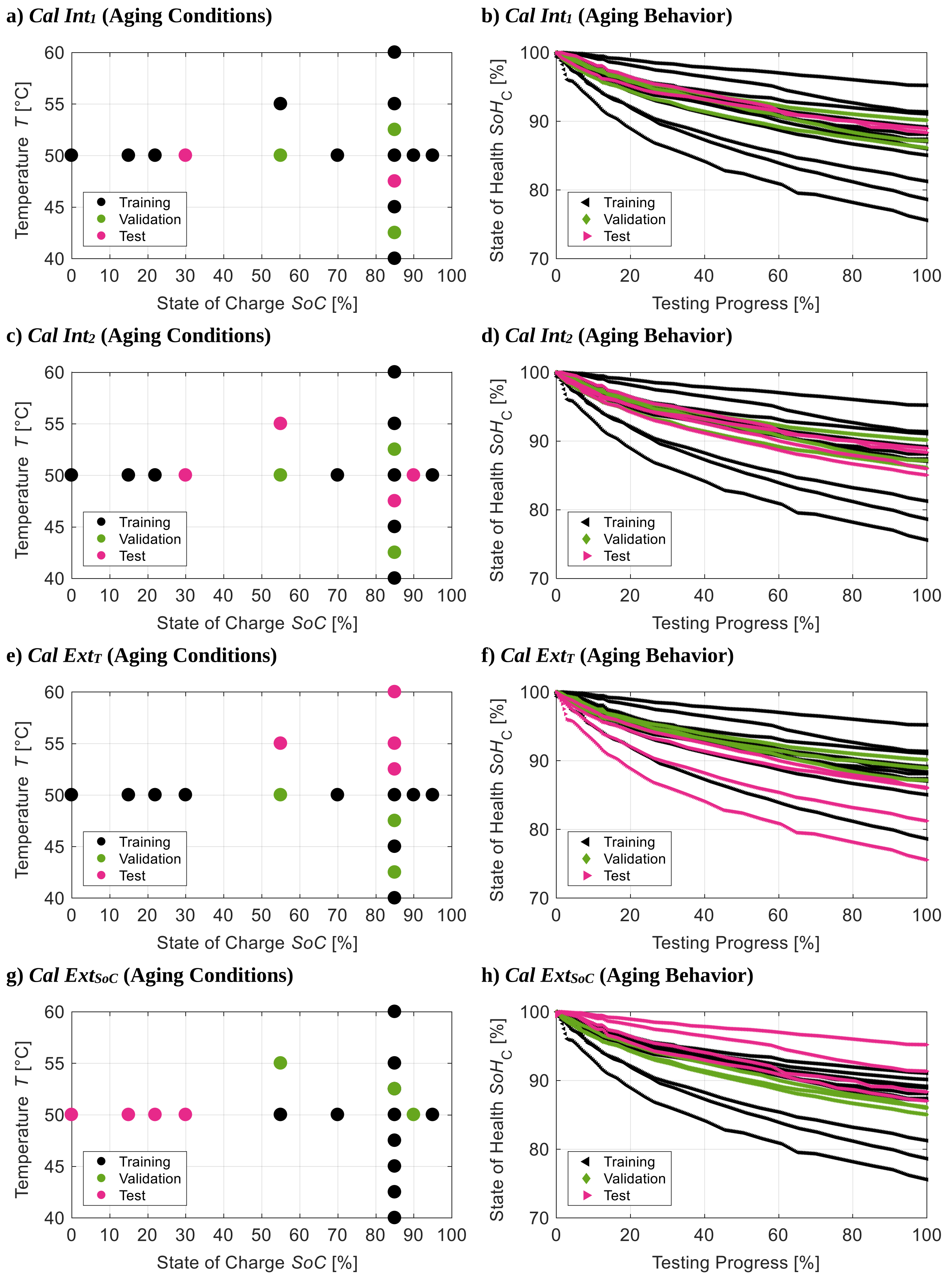

Figure S6. Calendar aging test cases investigating prediction over storage conditions: Aging conditions and corresponding aging behavior over storage time (=testing progress) of training, validation and test set of the test cases **a)/b)** *Cal Int*$_1$, **c)/d)** *Cal Int*$_2$, **e)/f)** *Cal Ext*$_T$ and **g)/h)** *Cal Ext*$_{SoC}$.

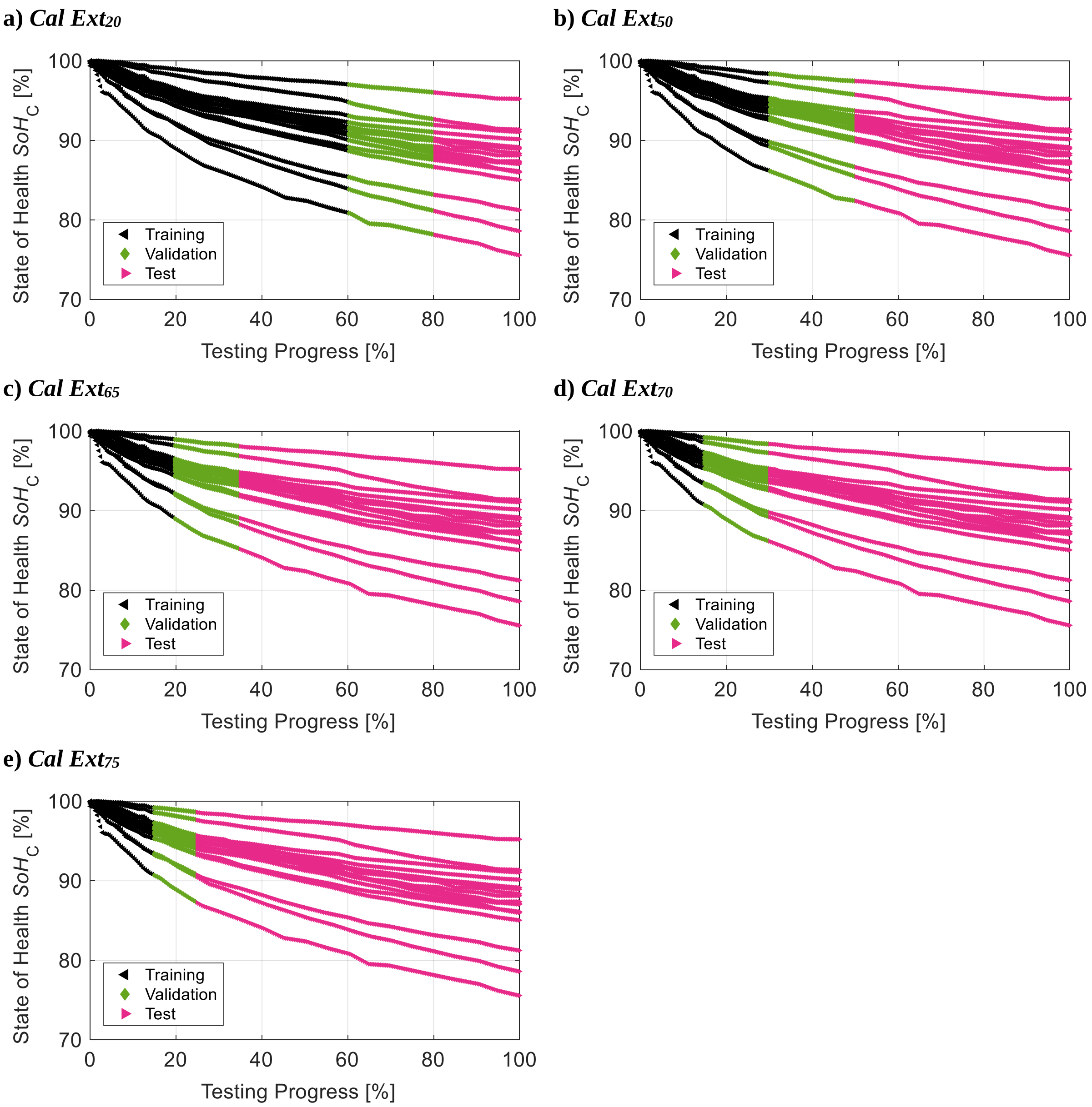

Figure S7. Calendar aging test cases investigating extrapolation over storage time (=testing progress): Training, validation and test set of the test cases **a)** *Cal Ext*$_{20}$, **b)** *Cal Ext*$_{50}$, **c)** *Cal Ext*$_{65}$, **d)** *Cal Ext*$_{70}$ and **e)** *Cal Ext*$_{75}$.

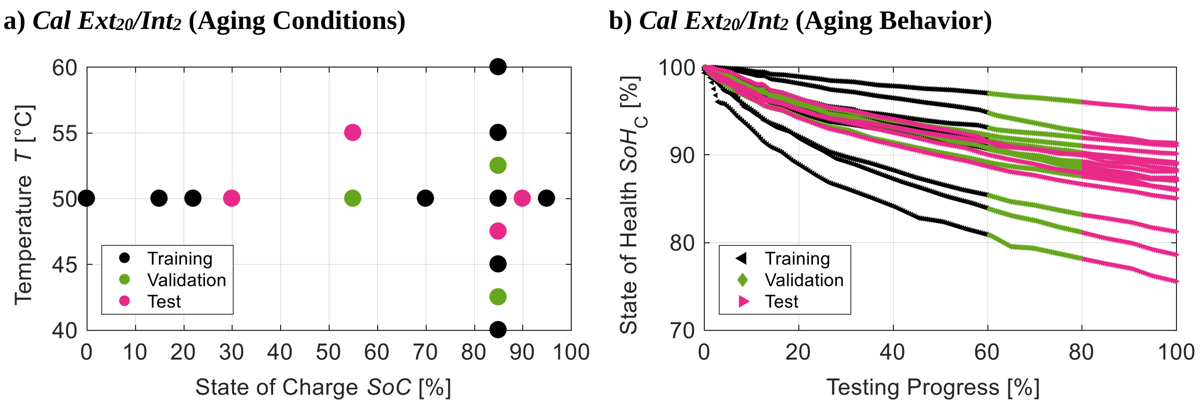

Figure S8. Calendar aging test case *Cal Ext*$_{20}$*/Int*$_{2}$ investigating combined prediction: a) Aging conditions and **b)** corresponding aging behavior over storage time (=testing progress) of training, validation and test set.

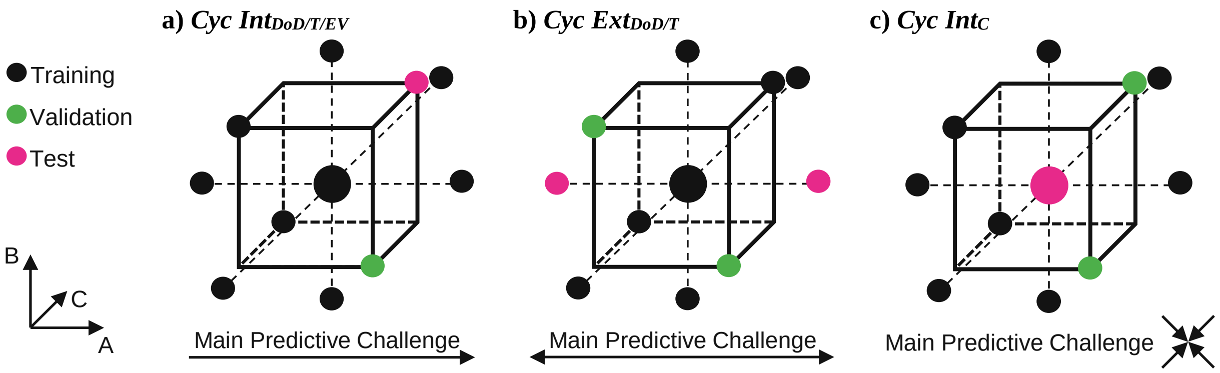

Figure S9. Principle of the partition by aging condition for cycle aging: The main predictive challenge can be influenced by the partition into training, validation and test set. The focus is put on correctly considering the influence of a specific stress factor for **a)** interpolation ($Cyc\ Int_{DoD}$, $Cyc\ Int_T$ and $Cyc\ Int_{EV}$) or **b)** extrapolation ($Cyc\ Ext_{DoD}$ and $Cyc\ Ext_T$) or on **c)** interpolating the center point ($Cyc\ Int_C$). For $Cyc\ Int_{DoD}$, $Cyc\ Int_T$ and $Cyc\ Int_{EV}$, the validation sets and test sets consist of five cubic points each. Since $Cyc\ Ext_{DoD}$ and $Cyc\ Ext_T$ investigate extrapolation over a specific stress factor, their test sets consist of four star points ($SoC_{\mathrm{min,min/max}}$, $SoC_{\mathrm{max,min/max}}$) for $Cyc\ Ext_{DoD}$ and two star points ($T_{\mathrm{min/max}}$) for $Cyc\ Ext_T$. The validation sets of $Cyc\ Ext_{DoD}$ and $Cyc\ Ext_T$ comprise six cubic points and eight cubic points, respectively. As $Cyc\ Int_C$ examines the capability to predict the center point, its test set only includes the center point. For its validation set, 50 % of the cubic points are randomly selected. For each test case, the remaining points make up the training set.

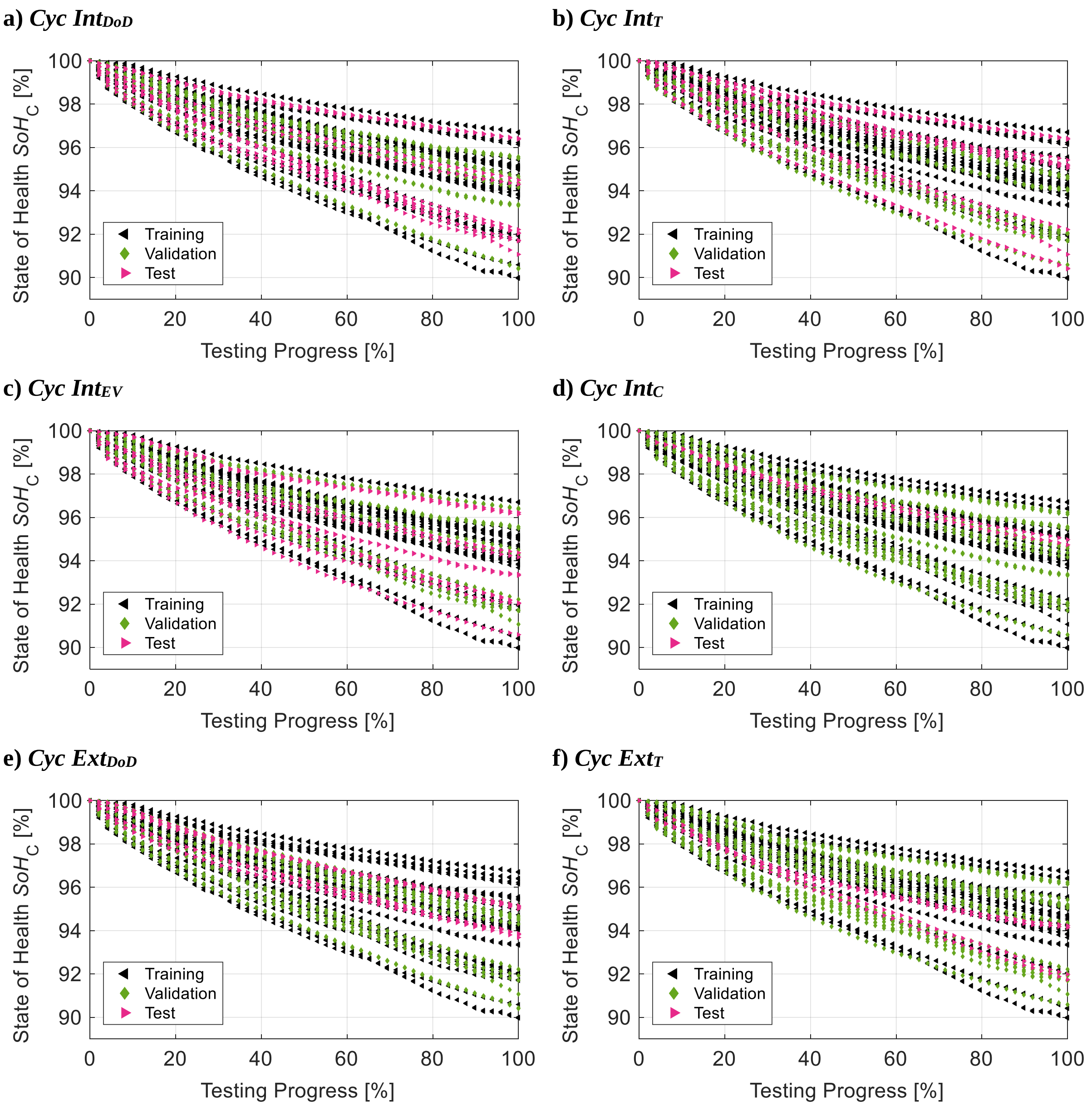

Figure S10. Cycle aging test cases investigating prediction over operating conditions: Aging behavior over energy throughput (=testing progress) of the aging conditions selected for training, validation and test set of the test cases **a)** *Cyc Int*$_{DoD}$, **b)** *Cyc Int*$_{T}$, **c)** *Cyc Int*$_{EV}$, **d)** *Cyc Int*$_{C}$, **e)** *Cyc Ext*$_{DoD}$ and **f)** *Cyc Ext*$_{T}$.

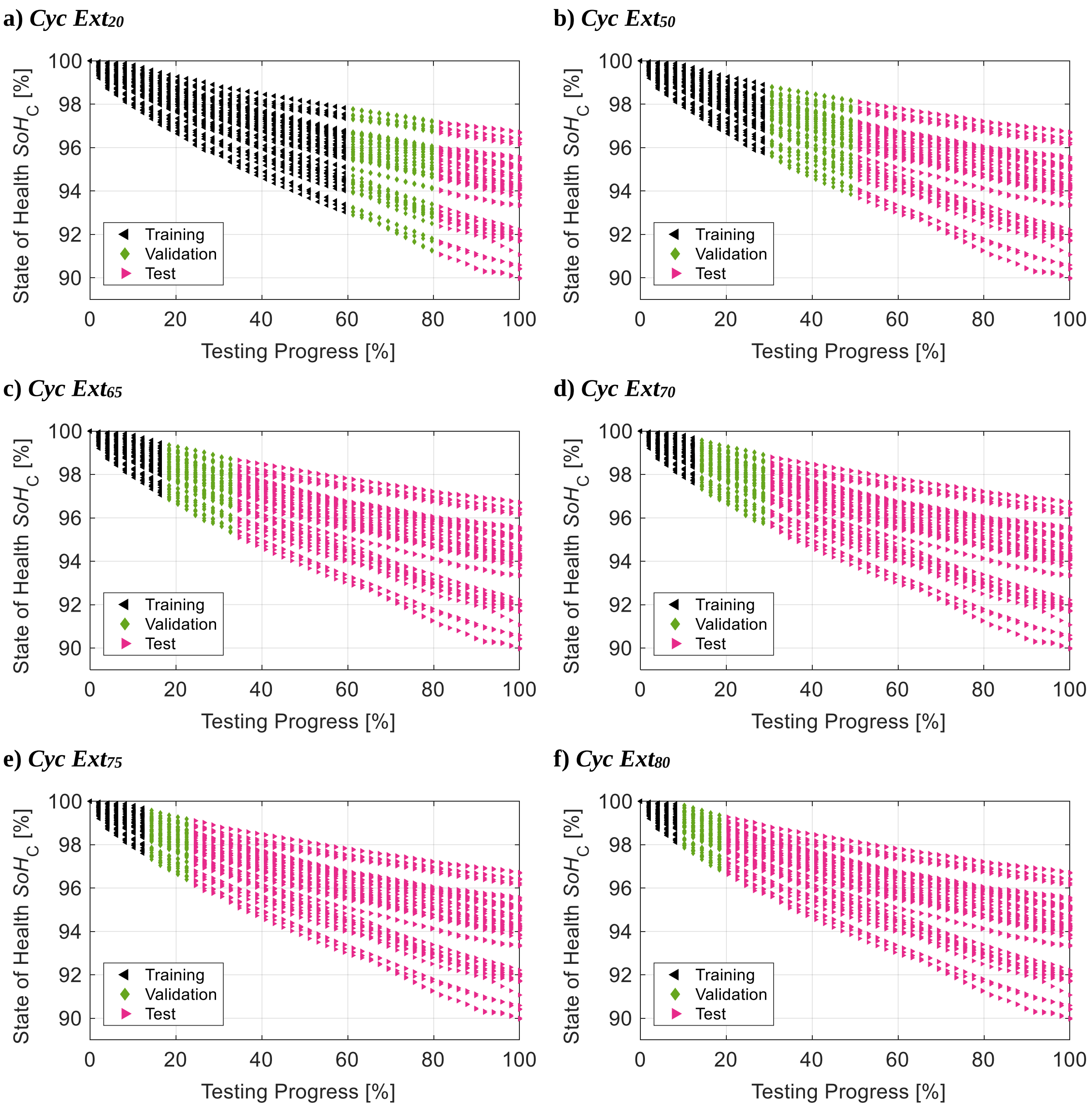

Figure S11. Cycle aging test cases investigating extrapolation over energy throughput (=testing progress): Training, validation and test set of the test cases **a)** *Cyc Ext$_{20}$*, **b)** *Cycl Ext$_{50}$*, **c)** *Cycl Ext$_{65}$*, **d)** *Cyc Ext$_{70}$*, **e)** *Cyc Ext$_{75}$* and **f)** *Cyc Ext$_{80}$*.

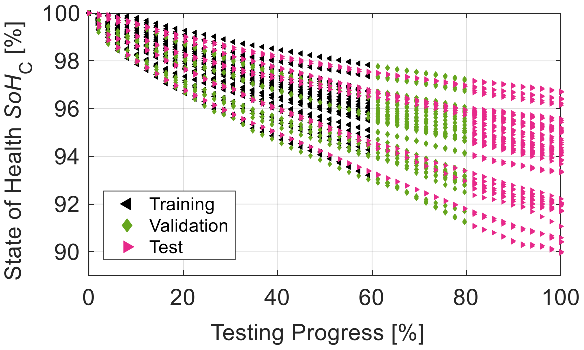

Figure S12. Cycle aging test case *Cyc Ext$_{20}$/Int$_{T}$* investigating combined prediction: Aging behavior over energy throughput (=testing progress) of the aging conditions selected for training, validation and test set.

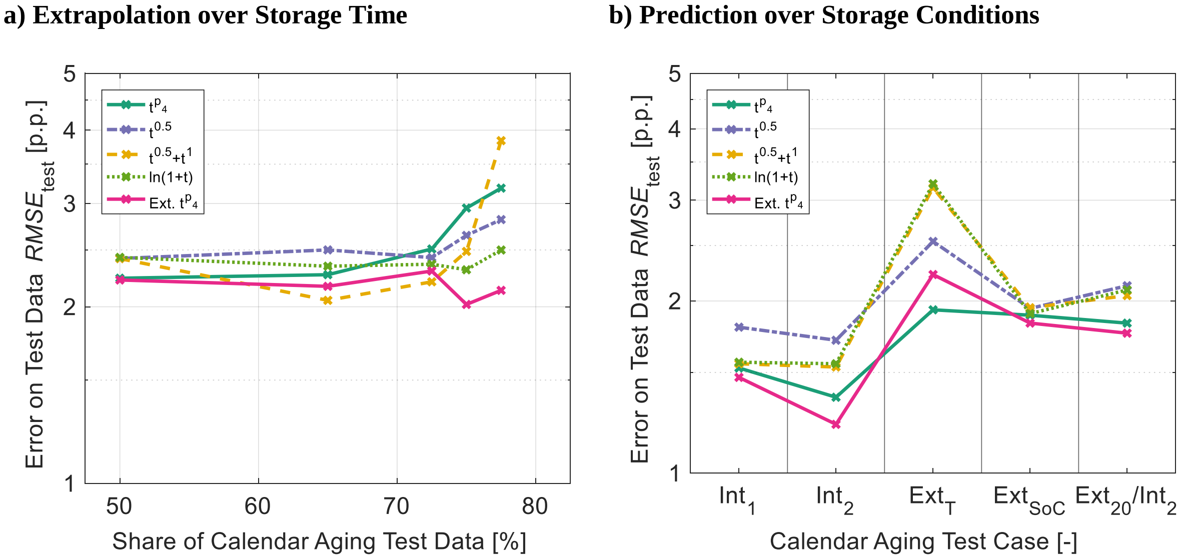

Figure S13. Comparison of (semi-)empirical calendar aging models: Investigation of **a)** extrapolation of calendar aging over storage time and **b)** prediction of calendar aging over storage conditions. The extended t^{p_4} model by Hahn et al.[10] is included to show improvements possible by including data from additional experiments.

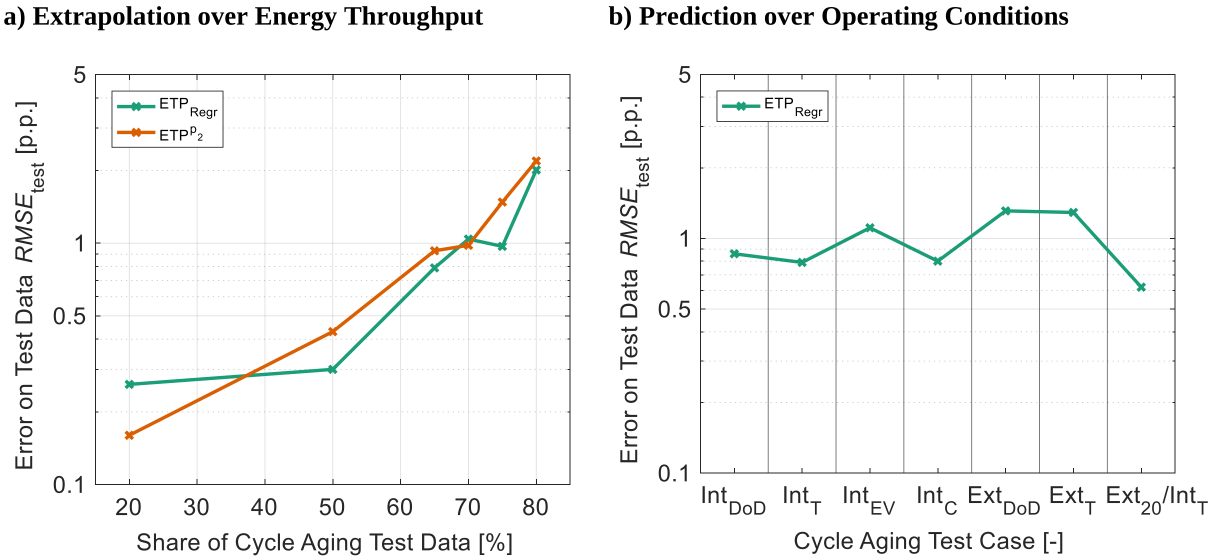

Figure S14. Comparison of (semi-)empirical cycle aging models: Investigation of **a)** extrapolation of cycle aging over energy throughput and **b)** prediction of cycle aging over operating conditions. The ETP^{p_2} model cannot make predictions over cell aging conditions.

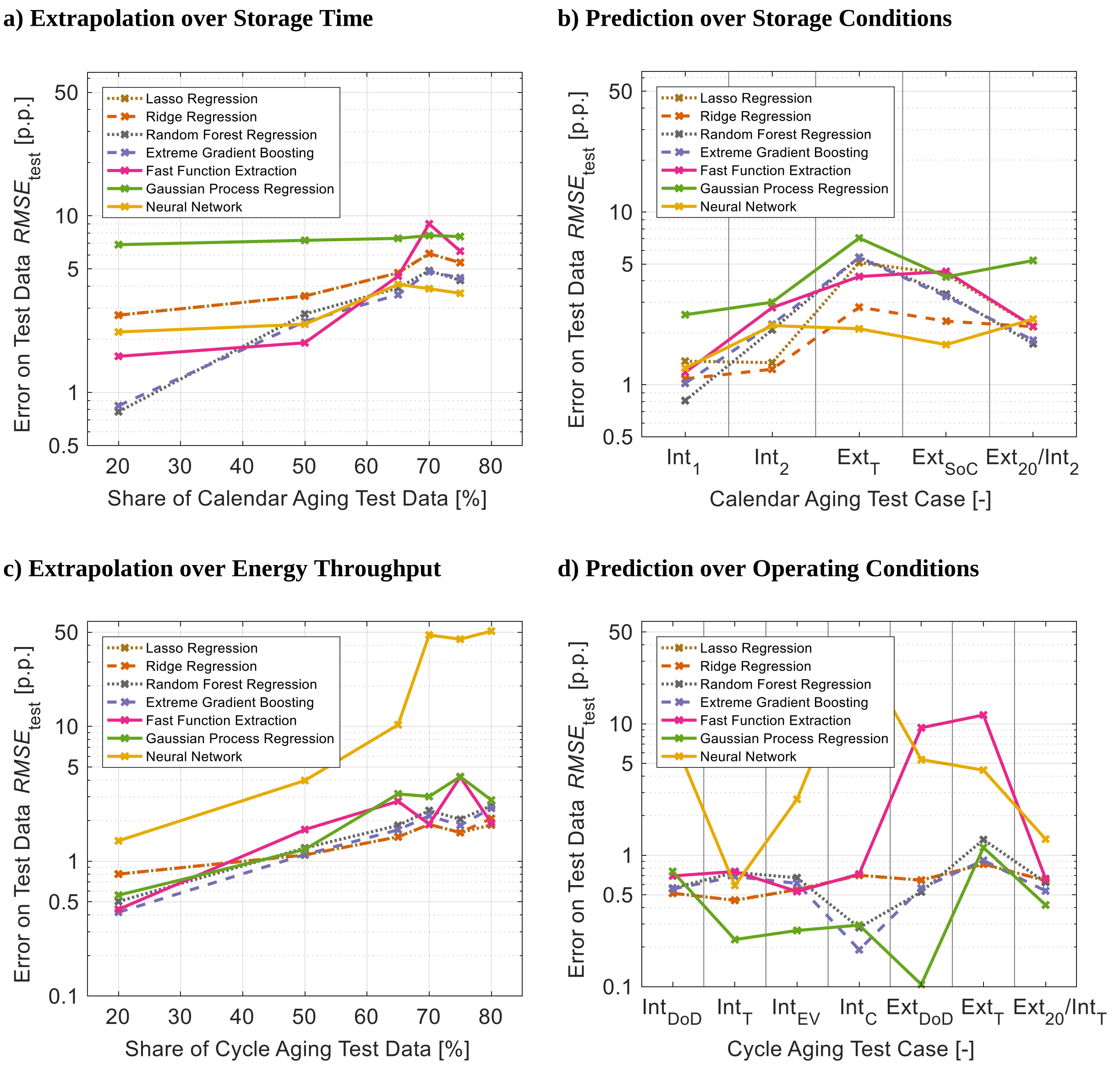

Figure S15. Comparison of machine learning approaches: Investigation of **a)** extrapolation of calendar aging over storage time, **b)** prediction of calendar aging over storage conditions, **c)** extrapolation of cycle aging over energy throughput and **d)** prediction of cycle aging over operating conditions.

a)
Variation of Aging Conditions

b)
Simulated Degradation for Constant Conditions

c)
Cumulative Degradation for Varying Conditions

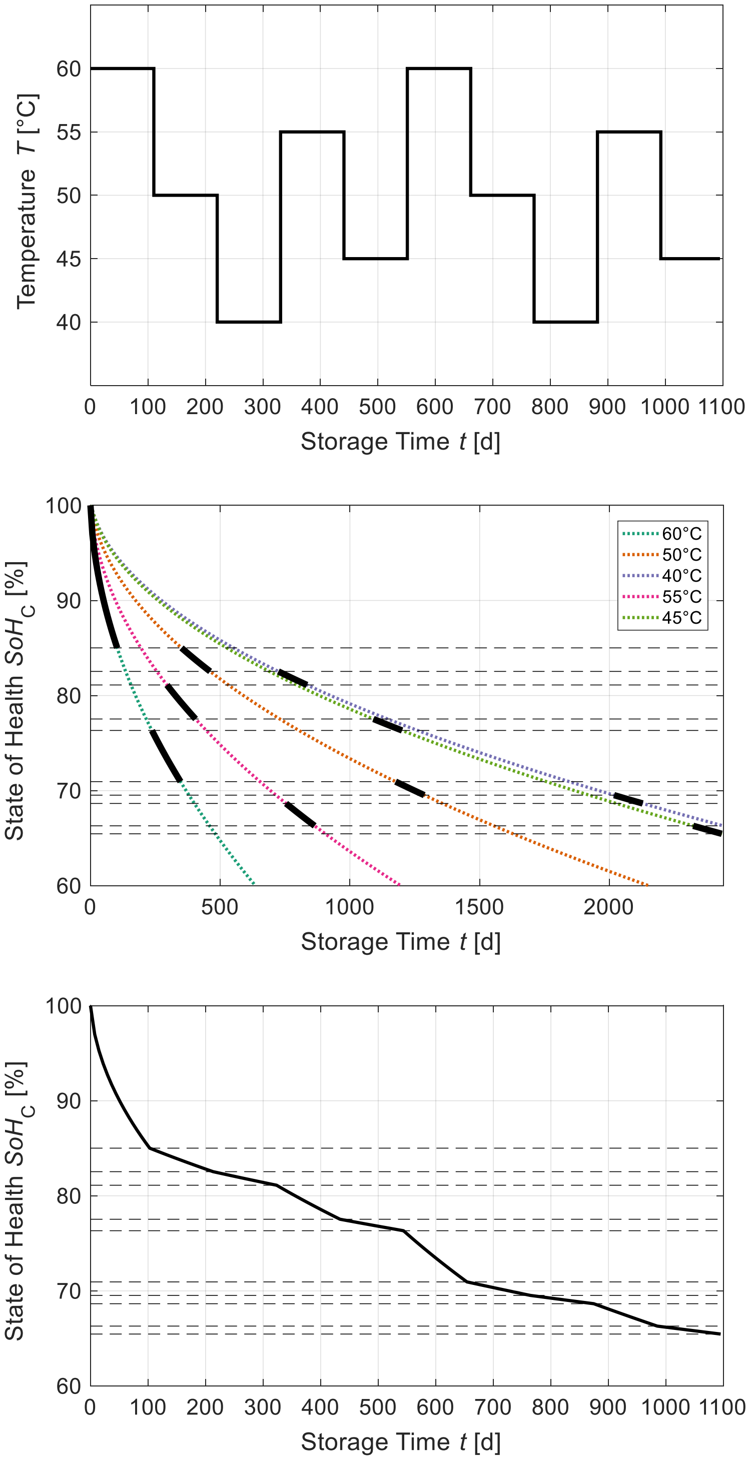

Figure S16. Cell degradation for varying aging conditions: For **a)** all aging conditions occurring in the investigation, **b)** degradation is simulated under constant conditions. Step-wisely adding parts of these degradation curves for each phase of constant aging conditions (highlighted in bold black), results in **c)** the cumulative degradation of the cell. At every change of aging conditions, a reference is calculated to determine the starting point on the degradation curve simulated for the conditions of the upcoming phase. This reference date $t_{ref,1}$ or reference energy throughput $ETP_{ref,1}$ indicates the amount of time or energy throughput necessary to reach the current total SoH_C under these new aging conditions. The corresponding ending point is determined by $t_{ref,2} = t_{ref,1} + \Delta t$ or $ETP_{ref,2} = ETP_{ref,1} + \Delta ETP$ with Δt / ΔETP between this and the next change of conditions.

a) ***Cal Ext*** $_{20}$

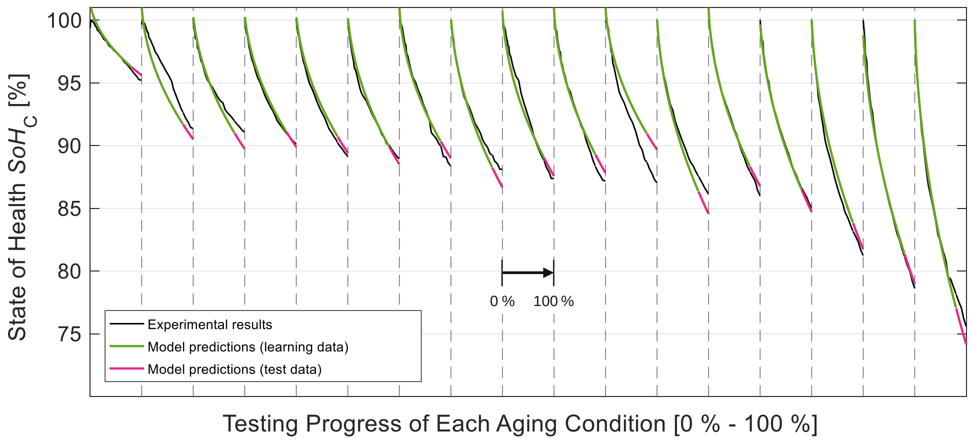

b) ***Cal Int*** $_{1}$

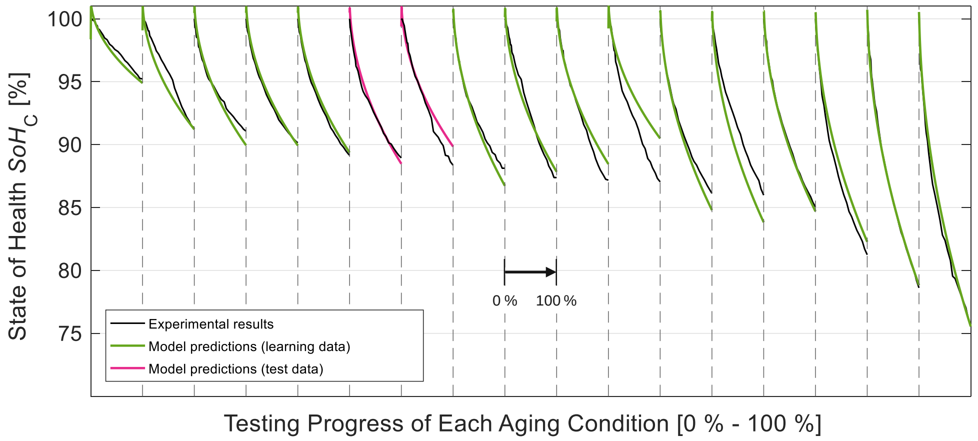

c) ***Cal Ext*** $_{SoC}$

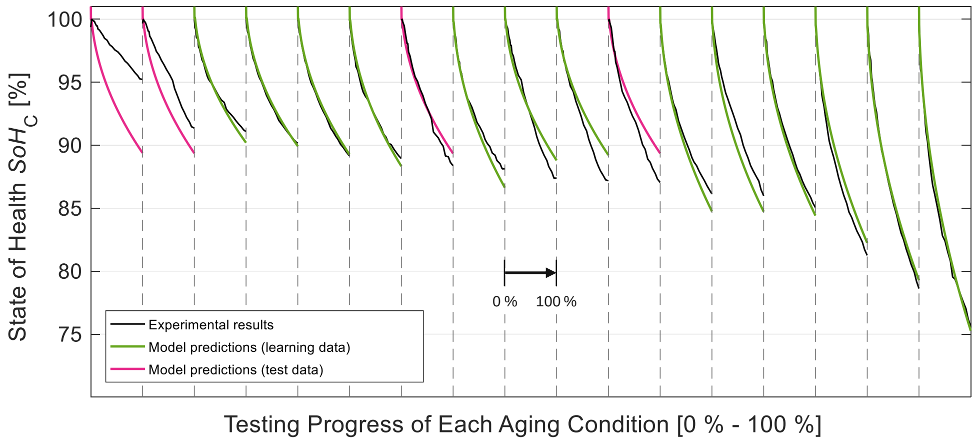

Figure S17. Comparison of model predictions with experimental results for selected calendar aging apex models: For a concise overview, apex models of the test cases **a)** *Cal Ext* $_{20}$, **b)** *Cal Int* $_{1}$ and **c)** *Cal Ext* $_{SoC}$ – and thus examples for every relevant task of lifetime prediction for calendar aging – are evaluated. Each test case's apex model represents general aging trends without describing noise.

a) ***Cyc Ext$_{80}$***

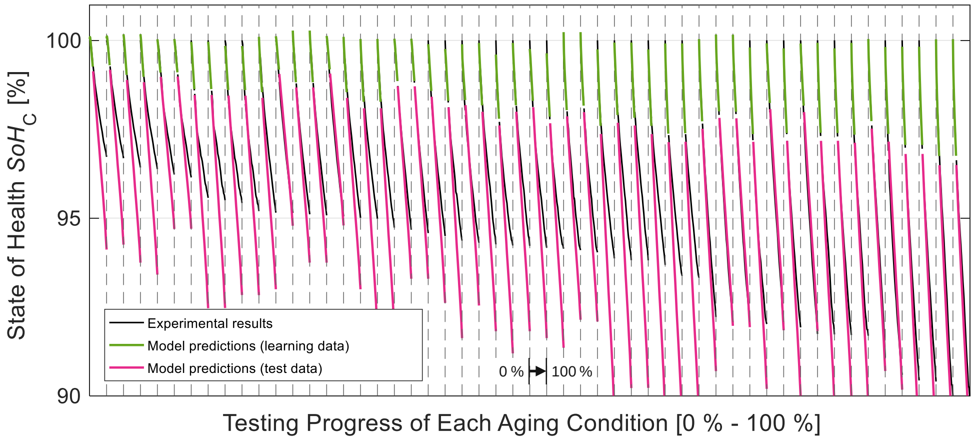

b) ***Cyc Int$_{DoD}$***

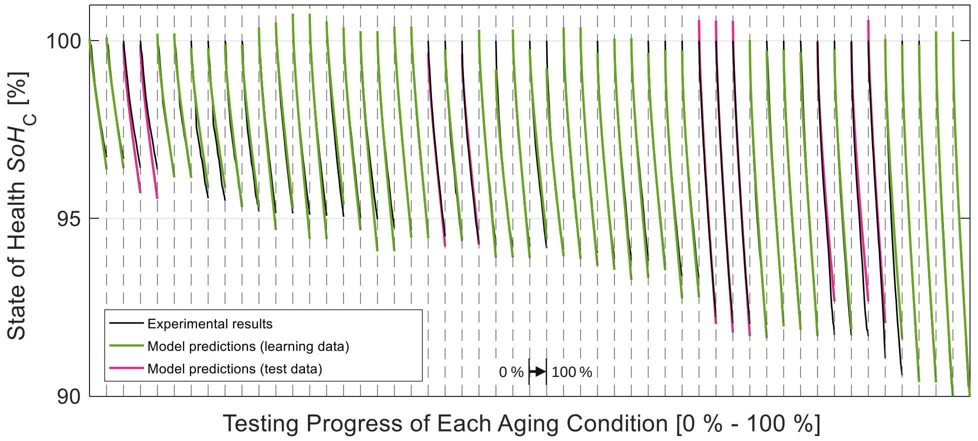

c) ***Cyc Ext$_T$***

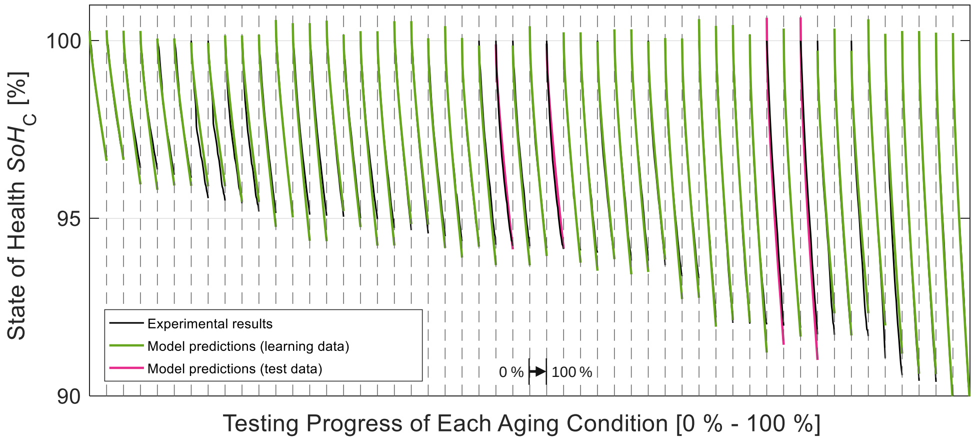

Figure S18. Comparison of model predictions with experimental results for selected cycle aging apex models: For a concise overview, apex models of the test cases **a)** *Cyc Ext$_{80}$*, **b)** *Cyc Int$_{DoD}$* and **c)** *Cyc Ext$_T$* – and thus examples for every relevant task of lifetime prediction for cycle aging – are evaluated. Each test case's apex model represents general aging trends without describing noise.

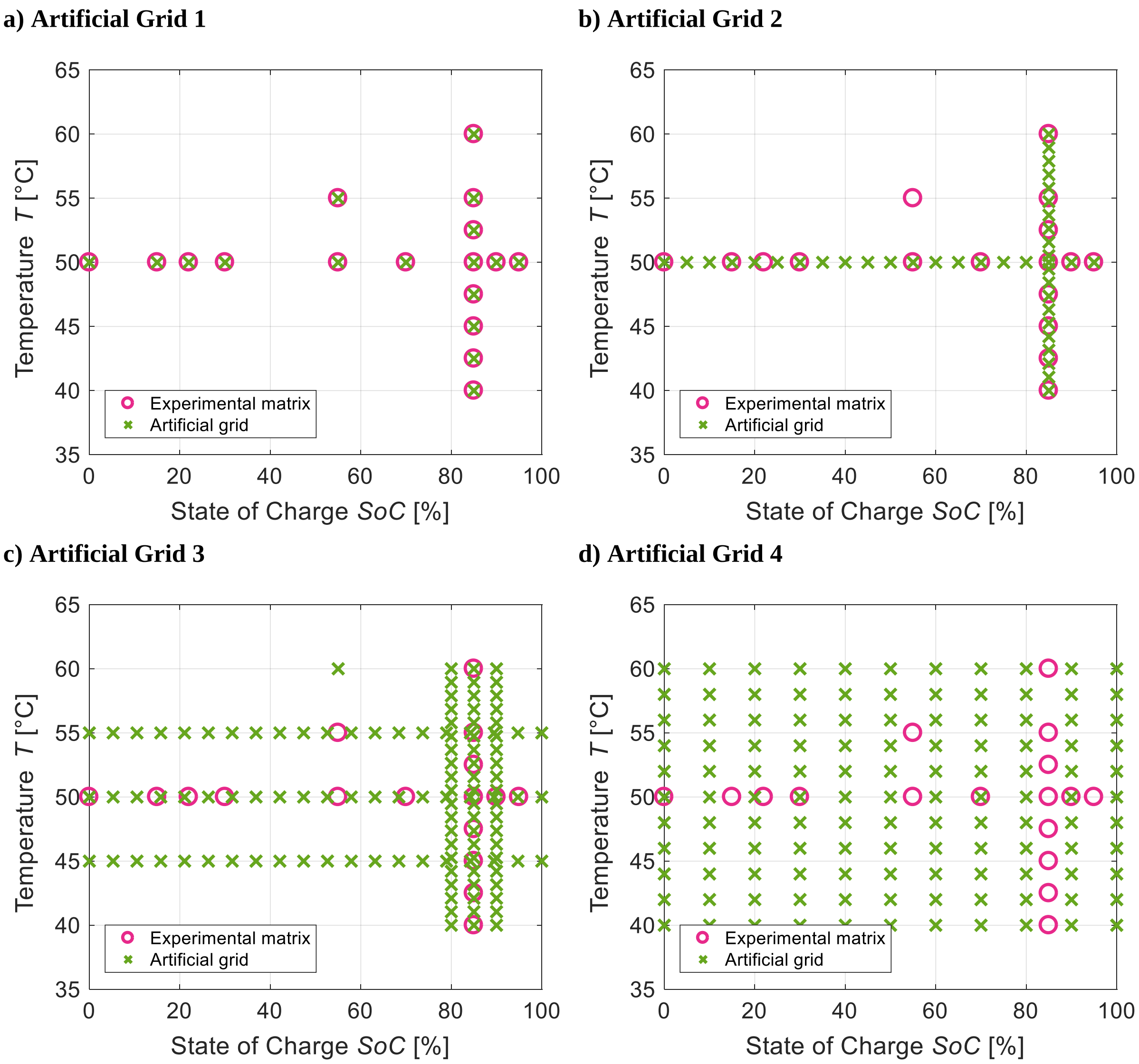

Figure S19. Artificial grids for hybridization: In a hybrid approach, evolutionary generated models are used to expand the learning data available for the parameterization of semi-empirical models. For this expansion of data, the apex model of the investigated test case is required to predict aging for multiple artificial grids - ranging from sole extrapolation over time to inter- and extrapolation over aging conditions in combination with extrapolation over time. **a)** Artificial grid 1 only requires extrapolation over storage time to the maximum storage time investigated by the test case (in this work's calendar aging case study: 280 days). Artificial grids 2-4 combine this task with predictions over storage conditions. **b)** Artificial grid 2 requires the interpolation of 20 equally distributed grid points per stress factor. **c)** Artificial grid 3 additionally includes extrapolation by extending artificial grid 2 with axes of grid points with offsets of $\Delta T = \pm 5$ K and $\Delta SoC = \pm 5$ p.p., respectively. **d)** Artificial grid 4 equally distributes data points across the entire range of each investigated stress factor. The expanded learning data is used to train the semi-empirical calendar aging benchmark model t^{p_4}. Best results can be achieved if the artificial grids are selected according to the task of the investigated test case: Artificial grid 1 for extrapolation over storage time, artificial grid 2 for interpolation between storage conditions and combined prediction and artificial grid 4 for extrapolation over storage conditions.

a) Learning Data

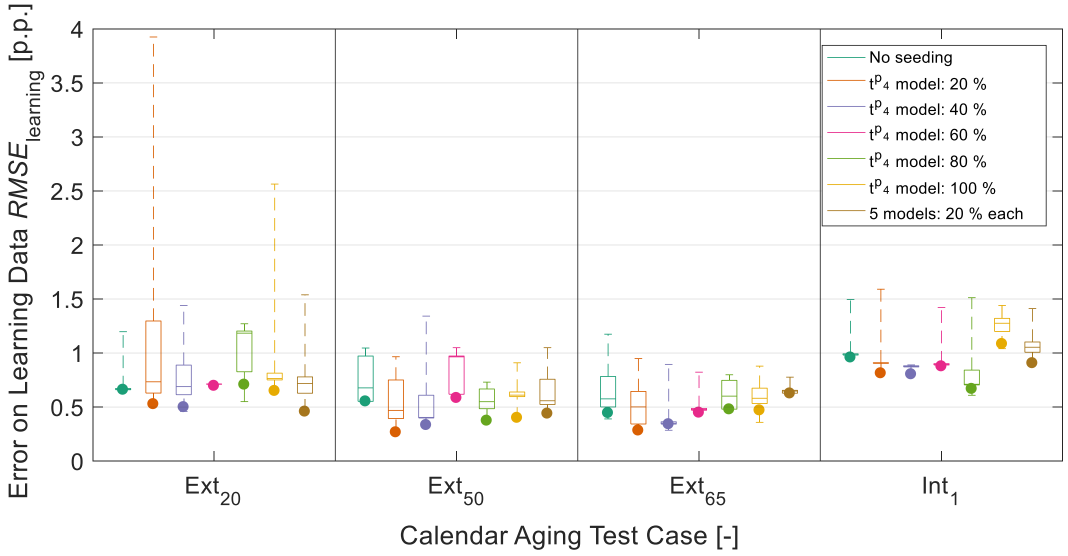

b) Test Data

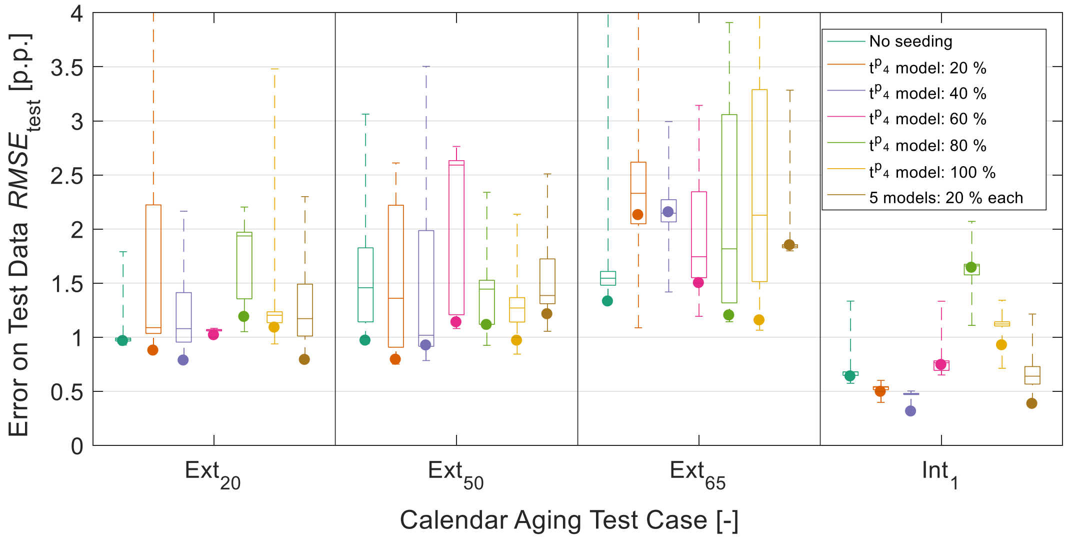

Figure S20. Comparison of different seeding approaches with random initialization of evolutionary processes in *Stage One*: For each approach, the median of all *Stage Two* champion models is visualized by the central mark of the box, whereas the 25th and 75th percentiles are indicated by the bottom and top edges, respectively. The whiskers represent extreme champions and the filled circle highlights the apex model. Evaluation on **a)** learning and **b)** test data.

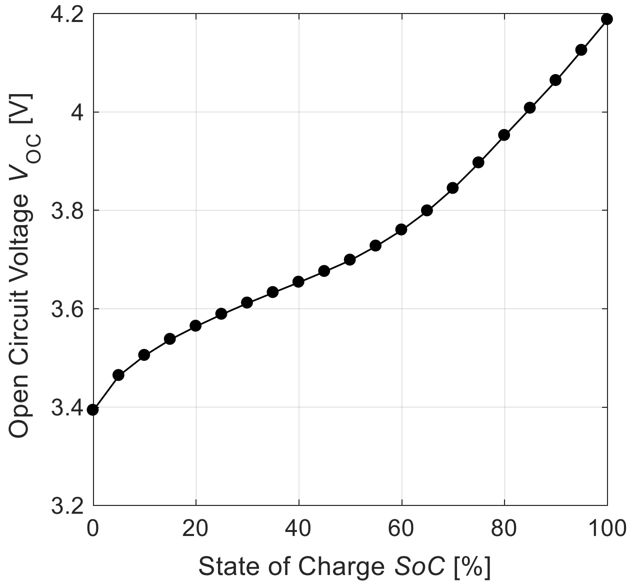

Figure S21. Correlation of *SoC* and open circuit voltage V_{OC} at begin of life and T = 25 °C: Usage for transformations between V_{cell} and *SoC* during states of equilibrium at any condition of the cells investigated in the calendar aging experiment.

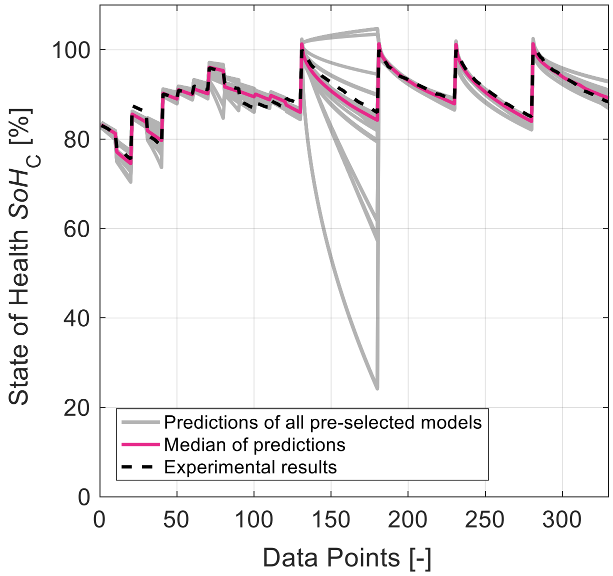

Figure S22. Principle of the crowd error $RMSE_{crowd}$: As exemplarily shown for *Cal* Ext_{20}/I_2, the median $\tilde{y}$ of the predictions of all pre-selected models resembles the trend of the test data's response variable despite not using this information. The SoH_C trend of the test data is discontinuous since it is a concatenation of data of multiple aging conditions with different degrees of aging and timeframes.

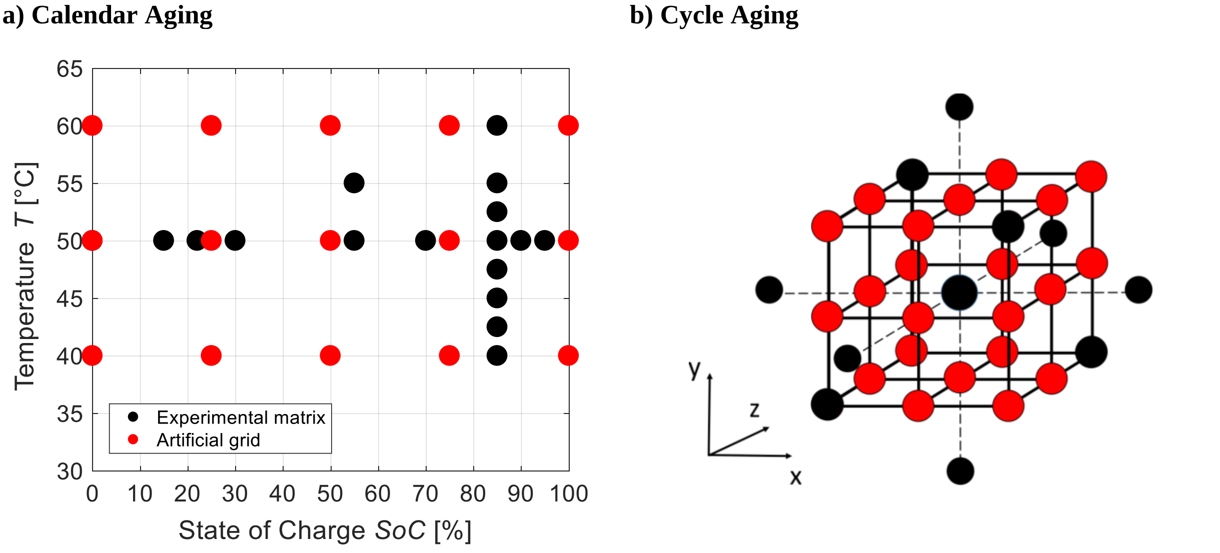

Figure S23. Design of artificial grids used in apex model selection: Comparison of experimental matrix and artificial grid for **a)** calendar aging and **b)** cycle aging (example with three factors x, y and z). The artificial grids enable an improved assessment of generalizability.

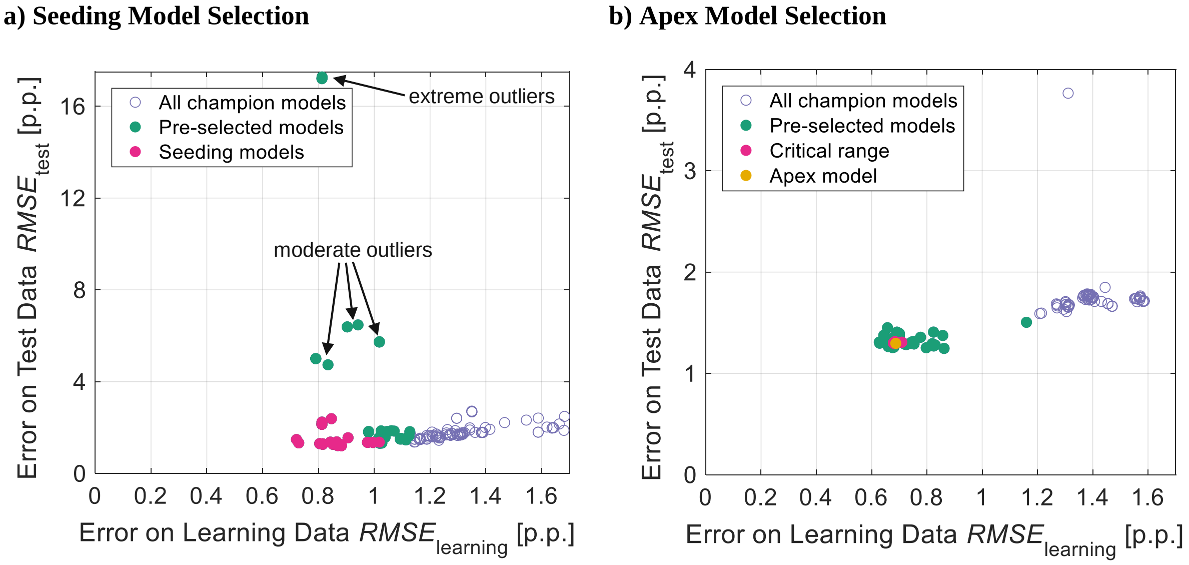

Figure S24. Stepwise progression of the model selection mechanisms for test case *Cal Ext₂₀/Int₂*: For **a)** seeding model selection in *Stage One* as well as **b)** apex model selection in *Stage Two*, only the best 40 % of champion models regarding $RMSE_{learning}$ are considered (pre-selection). Subsequently, these pre-selected models need to pass adaptive thresholds dependent on the crowd error $RMSE_{crowd}$. The remaining models (critical range) are evaluated with Equation 5 or Equation 6 to select 25 seeding models in *Stage One* or the apex model in *Stage Two*, respectively.

a) ***Fitness*₁** **b)** ***Fitness*₂**

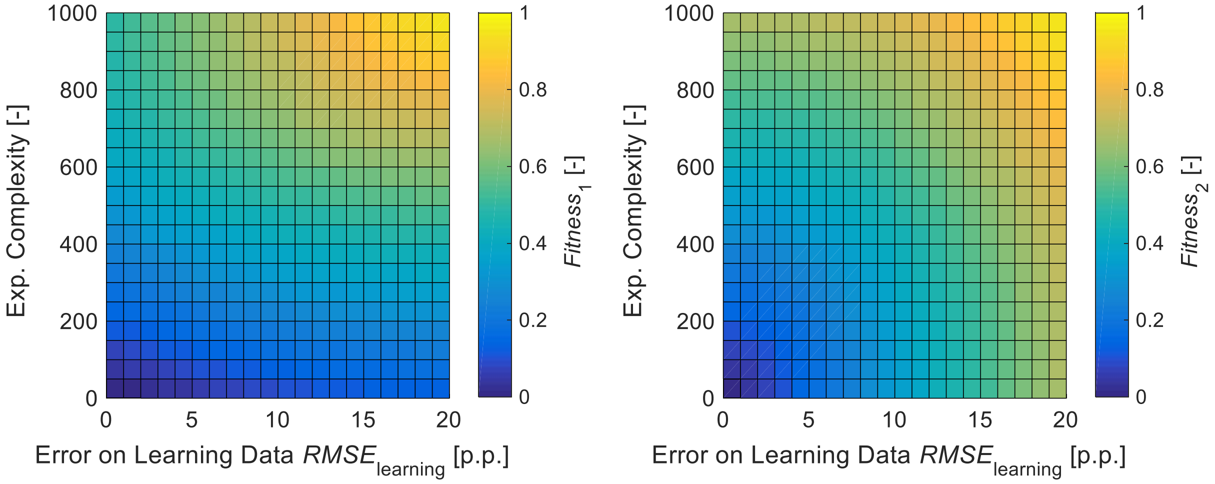

Figure S25. Areas of interest of multi-objective fitness functions for model evaluation: Functions for fitness evaluation define the area of interest of the optimization process they are used in. Therefore, it is desirable for the assumption of structure and weights of a scaled multi-objective fitness function to approximate the Pareto-front.[3] Our genetic programming framework uses two different scaled multi-objective fitness functions, which meet context specific requirements: **a)** $Fitness_1$ is used for model evaluation during and after each evolutionary process enabling parent and survivor selection as well as champion model selection. To guide the optimization towards generalizable models with rather simple and flat genes, the complexity term is given more weight than the error term. Consequently, the tolerated margin of error is substantially increased for low complexities. **b)** $Fitness_2$ is used for apex model and experiment selection. Since predominantly solutions of high quality take part in these selection processes, a narrower target range is required. For this, equal weights are applied for complexity and error term. This results in a reduced tolerance for high errors and complexities. Both functions require optimization by minimization.

Supporting Tables

Table S1. (Semi-)empirical models considered for the benchmark analysis of calendar aging models: The models are parameterized by the fitting parameters p_i. Dependencies derived from the physical equations by Arrhenius and Tafel are included in the terms $\exp(p_i/T)$ and $\exp(p_j \cdot V_{cell})$, respectively. The predictive performance of the models is compared by their $\overline{RMSE}$ averaged over all investigated calendar aging test cases. Even though recent advances were made by Hahn et al.[10] and Schimpe et al.[11], their models are not included in the benchmark analysis as these approaches require additional information obtained by specific begin of life experiments.

Abbreviation	Model	Reference	$\overline{RMSE}$ [p.p.]
$\boldsymbol{t^{p_4}}$	$SoH_C = 100 - p_1 \cdot e^{\frac{p_2}{T}} \cdot e^{p_3 \cdot V_{cell}} \cdot t^{p_4}$	Belt et al.[18]	2.1682
$\boldsymbol{t^{0.5}}$	$SoH_C = 100 - p_1 \cdot e^{\frac{p_2}{T}} \cdot e^{p_3 \cdot V_{cell}} \cdot t^{0.5}$	Ecker et al.[19]	2.2923
$\boldsymbol{t^{0.5} + t^1}$	$SoH_C = 100 - p_1 \cdot e^{\frac{p_2}{T}} \cdot e^{p_3 \cdot V_{cell}} \cdot t^{0.5} - p_4 \cdot e^{\frac{p_5}{T}} \cdot e^{p_6 \cdot V_{cell}} \cdot t^1$	Käbitz et al.[20]	2.2263
$\mathbf{ln}(\boldsymbol{1 + t})$	$SoH_C = 100 - p_1 \cdot e^{\frac{p_2}{T}} \cdot e^{p_3 \cdot V_{cell}} \cdot \ln(1 + p_4 \cdot t)$	Li et al.[21]	2.3230

Table S2. (Semi-)empirical models considered for the benchmark analysis of cycle aging models: The models are parameterized by the fitting parameters p_i. Superposed calendar aging effects are not subtracted from total aging. The model ETP_{Regr} comprises multiple models, each describing the SoH_C after a certain ETP. These models utilize linear, quadratic and simple interaction terms to take into account the influence of multiple stress factors SF_i. The predictive performance of the models is compared by their $\overline{RMSE}$ averaged over all investigated cycle aging test cases.

Abbreviation	Model	$\overline{RMSE}$ [p.p.]
$\boldsymbol{ETP^{p_2}}$	$SoH_C = 100 - p_1 \cdot ETP^{p_2}$	-
$\boldsymbol{ETP_{Regr}}$	$SoH_{C@ETP} = \sum_{i=1}^{n} [p_{i,1} \cdot SF_i + p_{i,2} \cdot {SF_i}^2 + \sum_{j=i+1}^{n} (p_{i,j} \cdot SF_i \cdot SF_j)]$	0.9346

Table S3. Machine learning approaches considered for the benchmark analysis: Overview of utilized software packages and hyperparameter search spaces. The predictive performance of the approaches is compared by their $\overline{RMSE}$ averaged over all investigated test cases.

Machine Learning Algorithm	Python Package	Hyperparameter Search Space	$\overline{RMSE}$ [p.p.]
**Lasso regression**	scikit-learn 0.22.1	alpha $\in [0, 50]$	2.1774
**Ridge regression**	scikit-learn 0.22.1	alpha $\in [0, 50]$	1.9816
**Random forest regression**	scikit-learn 0.22.1	max_depth $\in \{1, 2, \ldots, 20\}$<br>max_features $\in \{1, 2, \ldots, nr\_features\}$<br>n_estimators $\in \{100, 110, \ldots, 1000\}$	1.9761
**Extreme gradient boosting**	xgboost 1.1.1	n_estimators $\in \{20, 21, \ldots, 1000\}$<br>eta $\in \{0.025, 0.05, \ldots, 0.5\}$<br>max_depth $\in \{1, 2, \ldots, 14\}$<br>min_child_weight $\in \{1, 2, \ldots, 6\}$<br>subsample $\in \{0.5, 0.55, \ldots, 1\}$<br>gamma $\in \{0.1, 0.15, \ldots, 1\}$<br>colsample_bytree $\in \{0.5, 0.55, \ldots, 1\}$	1.9039
**Neural network (multi-layer perceptron)**	tensorflow 2.2.0	dropout rate hidden layer 1 $\in [0.01, \ldots, 0.5]$<br>dropout rate hidden layer 2 $\in [0.01, \ldots, 0.5]$<br>units hidden layer 1 $\in \{4, 20, \ldots, 256\}$<br>units hidden layer 2 $\in \{4, 20, \ldots, 256\}$<br>batch_size $\in \{16, 36, \ldots, 256\}$	3.2891
**Gaussian process regression**	scikit-learn 0.22.1	kernel = radial-basis function	3.3610
**Fast function extraction**	ffx 2.0.2	-	10.6157

Table S4. Calendar aging test cases: Tasks and share of data sets.

Test Case	Task	Share of Training Set [%]	Share of Validation Set [%]	Share of Test Set [%]
Cal $\boldsymbol{Int_1}$	Interpolation between storage conditions	70.6	17.6	11.8
Cal $\boldsymbol{Int_2}$	Interpolation between storage conditions	52.9	17.6	23.5
Cal $\boldsymbol{Ext_T}$	Extrapolation over *T*	52.9	17.6	23.5
Cal $\boldsymbol{Ext_{SoC}}$	Extrapolation over *SoC*	52.9	17.6	23.5
Cal $\boldsymbol{Ext_{20}}$	Extrapolation over *t*	60	20	20
Cal $\boldsymbol{Ext_{50}}$	Extrapolation over *t*	30	20	50
Cal $\boldsymbol{Ext_{65}}$	Extrapolation over *t*	20	15	65
Cal $\boldsymbol{Ext_{70}}$	Extrapolation over *t*	15	15	70
Cal $\boldsymbol{Ext_{75}}$	Extrapolation over *t*	15	10	75
Cal $\boldsymbol{Ext_{20}/Int_2}$	Extrapolation over *t* (*Cal* Ext_{20}) and interpolation between storage conditions (*Cal* Int_2)	35.3	29.4	35.3

Table S5. Cycle aging test cases: Tasks and share of data sets.

Test Case	Task	Share of Training Set [%]	Share of Validation Set [%]	Share of Test Set [%]
Cyc $\boldsymbol{Int_{DoD}}$	Interpolation between operating conditions with focus on the influence of *DoD*	61.5	19.2	19.2
Cyc $\boldsymbol{Int_T}$	Interpolation between operating conditions with focus on the influence of *T*	61.5	19.2	19.2
Cyc $\boldsymbol{Int_{EV}}$	Interpolation between operating conditions with focus on the influence of EV_{ratio}	61.5	19.2	19.2
Cyc $\boldsymbol{Int_C}$	Interpolation of the experiment's center point	65.4	30.8	3.8
Cyc $\boldsymbol{Ext_{DoD}}$	Extrapolation over *DoD*	61.5	23.1	15.4
Cyc $\boldsymbol{Ext_T}$	Extrapolation over *T*	61.5	30.8	7.7
Cyc $\boldsymbol{Ext_{20}}$	Extrapolation over *ETP*	60	20	20
Cyc $\boldsymbol{Ext_{50}}$	Extrapolation over *ETP*	30	20	50
Cyc $\boldsymbol{Ext_{65}}$	Extrapolation over *ETP*	20	15	65
Cyc $\boldsymbol{Ext_{70}}$	Extrapolation over *ETP*	15	15	70
Cyc $\boldsymbol{Ext_{75}}$	Extrapolation over *ETP*	15	10	75
Cyc $\boldsymbol{Ext_{80}}$	Extrapolation over *ETP*	10	10	80
Cyc $\boldsymbol{Ext_{20}/Int_T}$	Extrapolation over *ETP* (*Cyc* Ext_{20}) and interpolation between operating conditions with focus on the influence of *T* (*Cyc* Int_T)	37	31.5	31.5

Table S6. Comparison of (semi-)empirical benchmark model, machine learning benchmark approach and genetic programming apex models for calendar aging: Evaluation of calendar aging test cases by predictive error $RMSE_{\text{test}}$ and relative improvement of the genetic programming framework (GP) in comparison to the (semi-)empirical benchmark model t^{p_4} and the machine learning benchmark approach extreme gradient boosting (XG Boost).

Test Case	t^{p_4}: Predictive Error $RMSE_{\text{test}}$ [p.p.]	XG Boost: t^{p_4} Predictive Error $RMSE_{\text{test}}$ [p.p.]	GP: t^{p_4} Predictive Error $RMSE_{\text{test}}$ [p.p.]	GP vs. t^{p_4}: Relative Improvement [%]	GP vs. XG Boost: t^{i} Relative Improvement [%]
**Interpolation between storage conditions**					
Cal Int₁ → $Cal\ Int_1$	1.53	1.02	0.65	57.5	36.3
$Cal\ Int_2$	1.36	2.24	2.26	-66.2	-0.0
Average	-	-	-	**-4.3**	**17.7**
**Extrapolation over storage conditions**					
$Cal\ Ext_T$	1.93	5.44	2.64	-36.8	51.5
$Cal\ Ext_{SoC}$	1.89	3.25	2.63	-39.2	19.0
Average	-	-	-	**-38.0**	**35.3**
**Extrapolation over storage time**					
$Cal\ Ext_{20}$	2.24	0.84	0.97	56.7	-15.3
$Cal\ Ext_{50}$	2.27	2.52	1.02	55.1	59.6
$Cal\ Ext_{65}$	2.51	3.58	1.38	45.0	61.4
$Cal\ Ext_{70}$	2.95	4.82	1.81	38.6	62.5
$Cal\ Ext_{75}$	3.19	4.46	3.43	-7.5	23.1
Average	-	-	-	**37.6**	**38.3**
**Combined prediction**					
$Cal\ Ext_{20}/Int_2$	1.83	1.81	1.29	**29.5**	**28.8**

Table S7. Comparison of (semi-)empirical benchmark model, machine learning benchmark approach and genetic programming apex models for cycle aging: Evaluation of cycle aging test cases by predictive error $RMSE_{\text{test}}$ and relative improvement of the genetic programming framework (GP) in comparison to the (semi-)empirical benchmark model ETP_{Regr} and the machine learning benchmark approach extreme gradient boosting (XG Boost).

**Test Case**	**ETP_{Regr}: Predictive Error $RMSE_{\text{test}}$ [p.p.]**	**XG Boost: t^{p_4} Predictive Error $RMSE_{\text{test}}$ [p.p.]**	**GP: t^{p_4} Predictive Error $RMSE_{\text{test}}$ [p.p.]**	**GP vs. ETP_{Regr}: Relative Improvement [%]**	**GP vs. XG Boost: t^i Relative Improvement [%]**
**Interpolation between operating conditions**					
Cyc Int_{DoD}	0.86	0.55	0.46	46.5	17.0
Cyc Int_{T}	0.79	0.69	0.41	48.1	40.4
Cyc Int_{EV}	1.11	0.61	0.44	60.4	28.0
Cyc Int_{C}	0.80	0.19	0.26	67.5	-36.0
Average	-	-	-	**55.6**	**12.4**
**Extrapolation over operating conditions**					
Cyc Ext_{DoD}	1.31	0.56	0.40	69.5	28.9
Cyc Ext_{T}	1.29	0.91	0.30	76.7	67.2
Average	-	-	-	**73.1**	**48.1**
**Extrapolation over energy throughput**					
Cyc Ext_{20}	0.26	0.42	0.30	-15.4	28.3
Cyc Ext_{50}	0.30	1.12	0.41	-36.7	63.4
Cyc Ext_{65}	0.79	1.71	0.63	20.3	63.2
Cyc Ext_{70}	1.04	2.17	0.49	52.9	77.5
Cyc Ext_{75}	0.97	1.85	0.78	19.6	57.8
Cyc Ext_{80}	2.01	2.48	1.24	38.3	49.9
Average	-	-	-	**13.2**	**56.7**
**Combined prediction**					
Cyc Ext_{20}/Int_{T}	0.62	0.54	0.30	**51.6**	**44.0**

Table S8. Hyperparameters of the genetic programming algorithm considered in Bayesian optimization: A range of allowed values is defined for each hyperparameter in order to reduce the search space. During optimization, only values are considered, which are a multiple of the step size and do not exceed the limits specified by minimum and maximum.

Hyperparameter	Minimum	Maxi-mum	Step Size
**Group 0 – General**			
Population size [-]	100	1500	50
Number of generations [-]	100	1500	50
**Group 1 – Selection**			
Parent selection: Relative tournament size (compared to population size) [%]	5	50	5
Parent selection: Probability of using non-dominated sorting [%]	0	100	5
Survivor selection: Elite fraction (best individuals not replaced by offspring) [%]	0	50	5
**Group 2 – Reproduction**			
Probability of manipulation [%]	10	90	5
Probability of recombination [%]	10	90	5
Probability of cloning [%]	0	50	5
**Group 3 – Manipulation**			
Probability of subtree mutation [%]	10	100	5
Probability of point mutation type 1 [%]	0	20	2
Probability of point mutation type 2 [%]	10	80	5
Probability of point mutation type 3 [%]	0	10	1
Probability of point mutation type 4 [%]	0	10	1
Probability of point mutation type 5 [%]	0	10	1
Point mutation type 2 [%]: Maximum mutation in relation to the parent's value [%]	5	50	5
**Group 4 – Recombination**			
Probability of high level crossover [%]	0	100	20
Ratio of selected genes per parent [%]	0	100	20
**Group 5 – Genes**			
Maximum number of genes per individual [-]	1	5	1
**Group 6 – Trees**			
Maximum tree depth [-]	5	15	1
Maximum number of nodes per tree [-]	50	250	50
Maximum depth of mutation [-]	1	15	1

Supporting References

[1] D. P. Searson, in *Handb. Genet. Program. Appl.* (Eds.: A.H. Gandomi, A.H. Alavi, C. Ryan), Springer International Publishing, Cham, Germany, **2015**, pp. 551–573.

[2] M. Kommenda, Local Optimization and Complexity Control for Symbolic Regression, University Linz, **2018**.

[3] O. Kramer, *Genetic Algorithm Essentials*, Springer International Publishing, Cham, Germany, **2017**.

[4] A. E. Eiben, J. E. Smith, *Introduction to Evolutionary Computing*, Springer, Heidelberg, Germany, **2015**.

[5] L. Vanneschi, R. Poli, in *Handb. Nat. Comput.* (Eds.: G. Rozenberg, T. Bäck, J.N. Kok), Springer, Berlin, Germany, **2012**, pp. 709–739.

[6] Q. Lu, J. Ren, Z. Wang, *Comput. Intell. Neurosci.* **2016**, *2016*, 1.

[7] Y. Jin, B. Sendhoff, *IEEE Trans. Syst. Man, Cybern. Part C (Applications Rev.* **2008**, *38*, 397.

[8] A. Ghatak, *Machine Learning with R*, Springer, Singapore, Singapore, **2017**.

[9] F. Hutter, L. Kotthoff, J. Vanschoren, *Automated Machine Learning*, Springer International Publishing, Cham, Germany, **2019**.

[10] S. L. Hahn, M. Storch, R. Swaminathan, B. Obry, J. Bandlow, K. P. Birke, *J. Power Sources* **2018**, *400*, 402.

[11] M. Schimpe, M. E. von Kuepach, M. Naumann, H. C. Hesse, K. Smith, A. Jossen, *J. Electrochem. Soc.* **2018**, *165*, A181.

[12] J. Schmalstieg, S. Käbitz, M. Ecker, D. U. Sauer, *J. Power Sources* **2014**, *257*, 325.

[13] J. de Hoog, J.-M. Timmermans, D. Ioan-Stroe, M. Swierczynski, J. Jaguemont, S. Goutam, N. Omar, J. Van Mierlo, P. Van Den Bossche, *Appl. Energy* **2017**, *200*, 47.

[14] J. Wang, J. Purewal, P. Liu, J. Hicks-Garner, S. Soukazian, E. Sherman, A. Sorenson, L. Vu, H. Tataria, M. W. Verbrugge, *J. Power Sources* **2014**, *269*, 937.

[15] A. Cordoba-Arenas, S. Onori, Y. Guezennec, G. Rizzoni, *J. Power Sources* **2015**, *278*, 473.

[16] J. Bergstra, D. Yamins, D. Cox, in *Proc. 30th Int. Conf. Mach. Learn.* (Eds.: S. Dasgupta, D. McAllester), PMLR, Atlanta, Georgia, USA, **2013**, pp. 115–123.

[17] T. McConaghy, in *Genet. Program. Theory Pract. IX* (Eds.: R. Riolo, E. Vladislavleva, J.H. Moore), New York, NY, USA, **2011**, pp. 235–260.

[18] J. Belt, V. Utgikar, I. Bloom, *J. Power Sources* **2011**, *196*, 10213.

[19] M. Ecker, J. B. Gerschler, J. Vogel, S. Käbitz, F. Hust, P. Dechent, D. U. Sauer, *J.*

Power Sources **2012**, *215*, 248.

[20] S. Käbitz, J. B. Gerschler, M. Ecker, Y. Yurdagel, B. Emmermacher, D. André, T. Mitsch, D. U. Sauer, *J. Power Sources* **2013**, *239*, 572.

[21] D. Li, D. Danilov, Z. Zhang, H. Chen, Y. Yang, P. H. L. Notten, *J. Electrochem. Soc.* **2015**, *162*, A858.
